# Co-cultures of *Propionibacterium freudenreichii* and *Bacillus amyloliquefaciens* cooperatively upgrade sunflower seed milk to high levels of vitamin B_12_ and multiple co-benefits

**DOI:** 10.1186/s12934-022-01773-w

**Published:** 2022-03-26

**Authors:** Muzi Tangyu, Michel Fritz, Lijuan Ye, Rosa Aragão Börner, Delphine Morin-Rivron, Esther Campos-Giménez, Christoph J. Bolten, Biljana Bogicevic, Christoph Wittmann

**Affiliations:** 1 Institute of Systems Biotechnology, Saarland University, Saarbrücken, Germany; 2 Nestlé Research Center, Lausanne, Switzerland; 3 Nestlé Product Technology Center Food, Singen, Germany

**Keywords:** Sunflower seed milk, Co-culture, Microbial consortium, Vitamin B_2_, Vitamin B_3_, Vitamin B_7_, vitamin B_12,_l-lysine, Flavor, Indigestible sugar, *Propionibacterium freudenreichii* NCC 1177, *Bacillus amyloliquefaciens* NCC 156

## Abstract

**Background:**

Sunflower seeds (*Helianthus annuus*) display an attractive source for the rapidly increasing market of plant-based human nutrition. Of particular interest are press cakes of the seeds, cheap residuals from sunflower oil manufacturing that offer attractive sustainability and economic benefits. Admittedly, sunflower seed milk, derived therefrom, suffers from limited nutritional value, undesired flavor, and the presence of indigestible sugars. Of specific relevance is the absence of vitamin B_12_. This vitamin is required for development and function of the central nervous system, healthy red blood cell formation, and DNA synthesis, and displays the most important micronutrient for vegans to be aware of. Here we evaluated the power of microbes to enrich sunflower seed milk nutritionally as well as in flavor.

**Results:**

*Propionibacterium freudenreichii* NCC *1177* showed highest vitamin B_12_ production in sunflower seed milk out of a range of food-grade propionibacteria. Its growth and B_12_ production capacity, however, were limited by a lack of accessible carbon sources and stimulants of B_12_ biosynthesis in the plant milk. This was overcome by co-cultivation with *Bacillus amyloliquefaciens* NCC 156, which supplied lactate, amino acids, and vitamin B_7_ for growth of NCC 1177 plus vitamins B_2_ and B_3_, potentially supporting vitamin B_12_ production by the Propionibacterium. After several rounds of optimization, co-fermentation of ultra-high-temperature pre-treated sunflower seed milk by the two microbes, enabled the production of 17 µg (100 g)^−1^ vitamin B_12_ within four days without any further supplementation. The fermented milk further revealed significantly enriched levels of l-lysine, the most limiting essential amino acid, vitamin B_3_, vitamin B_6_, improved protein quality and flavor, and largely eliminated indigestible sugars.

**Conclusion:**

The fermented sunflower seed milk, obtained by using two food-grade microbes without further supplementation, displays an attractive, clean-label product with a high level of vitamin B_12_ and multiple co-benefits. The secret of the successfully upgraded plant milk lies in the multifunctional cooperation of the two microbes, which were combined, based on their genetic potential and metabolic signatures found in mono-culture fermentations. This design by knowledge approach appears valuable for future development of plant-based milk products.

**Supplementary Information:**

The online version contains supplementary material available at 10.1186/s12934-022-01773-w.

## Background

The world market of plant-based milk alternatives is rapidly increasing [1] and, notably, expected to surpass US$ 26 billion by 2023 [2]. Among various legumes and nuts, sunflower seeds (*Helianthus annuus*) emerge as attractive source for plant-based milk alternatives, given their high ecological sustainability, a natural richness in protein, magnesium and potassium, and a relatively low abundance in anti-nutritional factors and toxins [1, 3–5]. In addition, they are nut-free and suit consumers with celiac disease and food allergies. Of particular interest as raw material are the (defatted) press cakes of sunflower seeds, cheap residuals from sunflower oil manufacturing that offer attractive eco-friendliness and economic benefits [6]. It is therefore no surprise that sunflower seed suspensions (termed sunflower seed milk in this study due to their milk-like appearance) have found broad application as milk, yogurt, smoothies, and butter, among other products [6–8]. Admittedly, sunflower seed milk does not match animal milk in all desired characteristics, a drawback that is commonly reported for plant-based milk alternatives and requires upgrading [9].

One of the most severe limitations of plant-based products in general, and specifically sunflower seed milk, is the lack of vitamin B_12_ (cobalamin). Chemically, vitamin B_12_ is a cobalt-containing tetrapyrrole, one of the most complex small molecules made by nature [10]. It is crucial for neurodevelopment, cell division and cell differentiation, and displays the most important micronutrient for vegans to be aware of Ref. [11, 12]. Notably, only a few bacteria can make vitamin B_12_ [13]. Cobalt is found at the center of the molecule and is therefore ultimately required for biosynthesis [14]. In addition, the lower-ligand B_12_ precursor dimethylbenzimidazole (DMBI), its starter unit riboflavin (vitamin B_2_), nicotinamide (vitamin B_3_), catalytically involved in DMBI formation, and oxygen availability have been shown to support vitamin B_12_ production [14, 15], underlining the complexity of the biosynthetic process.

Other undesired features of sunflower seed milk are low levels of essential amino acids, especially l-lysine [16], the presence of indigestible sugars such as stachyose and raffinose [17], and a bitter, seedy taste [18, 19]. To overcome B_12_ limitation in plant-based materials, direct supplementation of limiting ingredients is a common way [20, 21]. However, this adds substantial extra costs, and does no longer meet the expectations of consumers, who more and more expect naturally derived, clean-label food and beverages without artificial blending [2]. At this point, fermentation offers an appealing option to naturally increase nutritional quality and taste of plant-based milk. In particular, mono-culture fermentation of plant-based materials is well established and has been used to increase e.g. vitamin B_12_ levels [22–24]. Often, however, fermentative synthesis of the vitamin is enabled by undesired non-natural supplementation of precursors and supporting agents [1, 24–27].

In this work, we developed a novel co-culture process for natural fermentation of sunflower seed milk to increase the vitamin B_12_ level. An initial screening round identified potential strains of *Propionibacterium freudenreichii*, which displays the only generally regarded as safe (GRAS) approved bacterium, known to synthesize active B_12_ [22], for fermentative production of the vitamin in sunflower seed milk. Subsequently, metabolic activities of the best performing strain *P. freudenreichii* NCC 1177 were analysed and provided a detailed signature profile. *Bacillus amyloliquefaciens* NCC 156 and *Lactocaseibacillus paracasei* subsp. *paracasei* NCC 2511, two microbes recently found well-performing in plant milk fermentation [28], revealed rather complementary features suggesting to co-culture them with the *Propionibacterium* to promote its growth and vitamin B_12_ biosynthetic power. After iterative optimization rounds, co-cultures of *P. freudenreichii* NCC 1177 and *B. amyloliquefaciens* NCC 156 were identified as the best set up. Co-cultivation of the two strains in sunflower seed milk increased the level of vitamin B_12_ up to 17 µg (100 g)^−1^, almost eight-fold more than initially observed for the *Propionibacterium* alone. Furthermore, the co-culture increased the levels of vitamins B_3_ and B_6,_ and the essential amino acid l-lysine, improved the protein digestibility corrected amino acid score (PDCAAS), decreased indigestible carbohydrate levels, and improved flavor. The developed co-culture approach seems valuable to upgrade sunflower seed milk and potentially also other plant milk materials.

## Results

### Evaluation of food-grade propionibacteria for their vitamin B_12_ production capacity

**Fig. 1:**
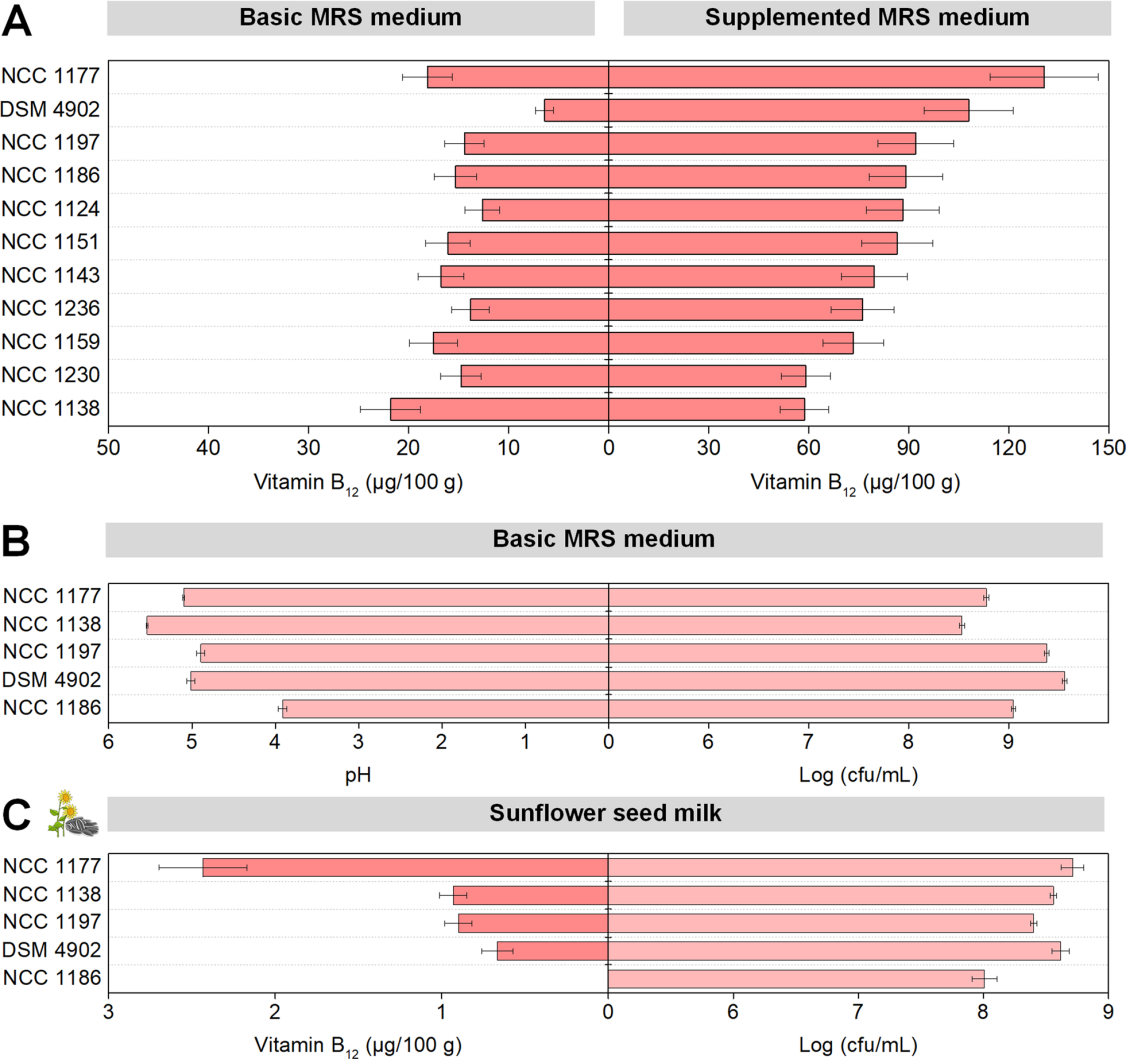
Screening of food-grade strains of *P. freudenreichii* for vitamin B_12_ synthesis. The data comprise the final titer of vitamin B_12_ in basic MRS medium and in MRS medium, supplemented with 50 µM CoCl_2_ and 100 µM dimethylbenzimidazole (DMBI) (**A**). In addition, pH, and growth of selected *P. freudenreichii* strains in MRS medium (**B**), and vitamin B_12_ production and growth of selected *P. freudenreichii* strains in pasteurized sunflower seed milk medium is shown (**C**). *P. freudenreichii* DSM 4902 was used as a positive control. The process was carried out as dual phase cultivation with an initial anaerobic phase (48 h), followed by an aerobic phase (24 h). n = 3

In an initial round of experiments, food-grade strains of *P. freudenreichii* were screened in MRS medium for their suitability to synthetize and secrete vitamin B_12_. Because anaerobic conditions seemed crucial to grow the cells [29], while aerobic conditions had proven beneficial to support vitamin B_12_ biosynthesis [30], dual phase cultures were conducted. After two days of anaerobic fermentation, the cells were shifted to aerobic conditions for another day. All strains formed the desired vitamin (Fig. 1A), whereby the B_12_ level ranged between 6.4 and 21.8 µg (100 g)^−1^. Vitamin B_12_ production in MRS medium was enhanced up to almost 150 µg (100 g)^−1^ by the addition of CoCl_2_ (50 µM) and DMBI (100 µM). Interestingly, the strains differed quite substantially in the extent, to which B_12_ production was activated by the stimulant mixture. Based on the data, five strains appeared most interesting: *P. freudenreichii* NCC 1177 (the best producer in supplemented MRS medium), NCC 1138 (the best producer in basic MRS medium), NCC 1197 and NCC 1186 (two strains with pronounced activation potential), and *P. freudenreichii* DSM 4902 (previously reported to be a vitamin B_12_ producer) [22].

To better understand the needs and capabilities of these strains, we assessed cell growth, residual glucose, and final pH value. It turned out that the strains differed in glucose utilisation. Four strains did not completely use up the sugar (1.04 g L^−1^ contained in the MRS medium) but left over a fraction of 37% (NCC 1177), 40% (DSM 4902), 43% (NCC 1197), and even 70% (NCC 1138). This limited capacity was likely due to an inhibiting acidification of the medium to a pH value around 4.9 – 5.5 (Fig. 1B). In contrast, strain NCC 1186 appeared more acid tolerant. Its medium pH dropped to 3.9 while glucose was fully depleted which corresponded to the fact that this strain reached the highest cfu number. Taken together, it seemed that the observed differences in vitamin B_12_ production were not simply due to differences in growth. As example, NCC 1186, yielded much less of the vitamin than NCC 1177, despite better pH tolerance, sugar uptake, and growth. Based on the data, the five strains were taken further to assess their capability to produce vitamin B_12_ in sunflower seed milk.

### Sunflower seed milk composition

**Fig. 2:**
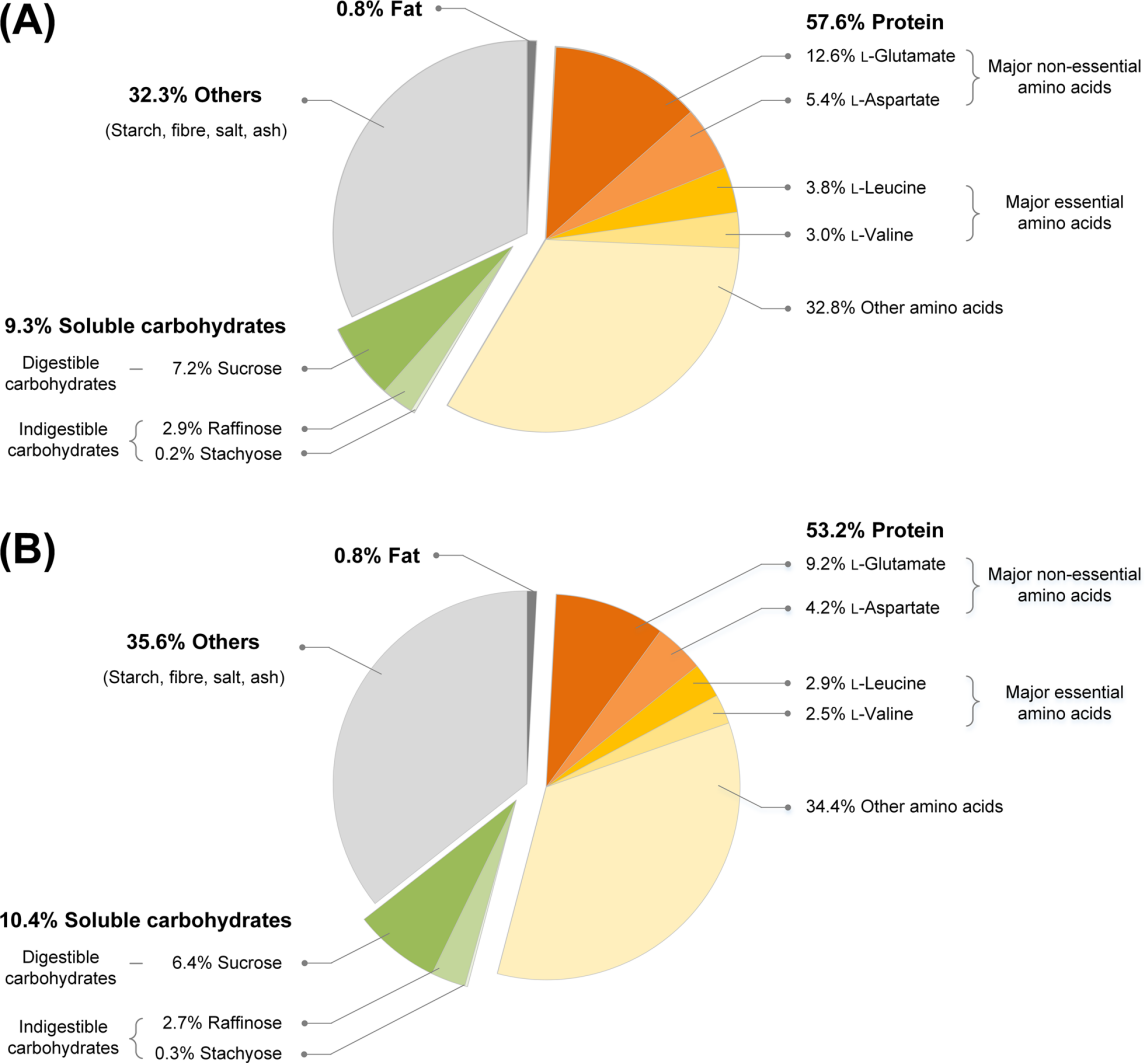
Composition of lab-scale pasteurized sunflower seed milk and ultra-high-temperature processed sunflower seed milk. All values are related to the dry mass (w/w). n = 3

Sunflower seed milk was prepared as aqueous solution of defatted sunflower seed flour. Batches at small scale were prepared by sterilization of the milk using low-pressure-pasteurization (LPP). This procedure provided a sterile, homogenous suspension. Protein represented the largest fraction (57.6% w/w dry mass), followed by soluble carbohydrates (9.3%), and only a low share of fat (0.8%) (Fig. 2A). The remaining fraction (32.3%) was attributed to e. g. fibres, ash, and salt. The most abundant non-essential amino acids were L-glutamate (12.6%) and L-aspartate (5.4%). The major essential amino acids were L-leucine (3.8%) and L-valine (3.0%). Soluble carbohydrates comprised digestible (sucrose) and indigestible sugars (raffinose, stachyose). Hereby, sucrose (7.2%) and raffinose (2.9%) showed the highest level, while stachyose was present in low amount (0.2%). L-Lysine was the most limiting essential amino acid. Related to the protein content, the level of L-lysine was 40 mg (g protein)^−1^, which represented only 69% of the FAO recommended value for children of 58 mg (g protein)^−1^ [31].

In addition, the milk was also ultra-pasteurized at ultrahigh temperature and pressure to represent industrially preferred treatment [32]. We therefore processed a 50 kg batch of UHT sunflower seed milk at pilot scale, including pre-warming to 75 °C, heating to 143 °C for 4 s, followed by efficient cooling. Overall, the chemical composition of UHT sunflower seed milk was comparable to that of LPP sunflower seed milk (Fig. 2B). Visual inspection revealed a less brownish color of the UHT plant milk, indicating less pronounced Maillard reactions by the shorter heating.

### *P. freudenreichii* NCC 1177 performs best in sunflower seed milk among a panel of tested strains

The five most promising strains from the screening (Fig. 1A, B) were now evaluated in the milk. When cultivated in sunflower seed milk for three days—two days under anaerobic conditions, followed by one day under aerobic conditions—strain NCC 1177 showed by far the best performance (Fig. 1C). The microbe produced 2.4 µg (100 g)^−1^ vitamin B_12_. In quantitative terms, NCC 1177 accumulated 160% more of the vitamin than NCC 1138, the second-best strain, and up to threefold more than the other three strains. NCC 1177 was also the best grower, as indicated by the high level of living cells. For NCC 1186, weak growth appeared somewhat linked to insignificant vitamin B_12_ formation. The other three strains were medium growers and medium B_12_ producers. The observed differences indicated that growth and B_12_ production in the milk were linked. As example, NCC 1177 produced 2.5-fold more vitamin B_12_ than NCC 1197, while reaching a twofold higher cfu number. This observation suggested that good growth in the milk seemed, at least, one prerequisite for high vitamin B_12_ levels. The best-performing strain, NCC 1177, was selected for further studies.

### Vitamin B_12_ biosynthesis of ***P. freudenreichii*** NCC 1177 in sunflower seed milk is limited by a lack of accessible carbon sources and B_12_ precursors

Next, we elucidated the potential to stimulate vitamin B_12_ production of strain NCC 1177 in sunflower seed milk. We tested supplementation with cobalt and DMBI, given their positive effects on vitamin B_12_ synthesis in MRS medium (Fig. 1A). Furthermore, we tested a range of other supporting ingredients, namely the DMBI precursor riboflavin (vitamin B_2_), nicotinamide (vitamin B_3_), previously shown to catalytically enhance DMBI formation [15], different amino acids, incorporated during vitamin B_12_ biosynthesis (l-threonine, l-glutamate, and glycine), and succinate as donor for succinyl-CoA at the start of the B_12_ pathway. In addition, glucose and lactate were tested as carbon sources to stimulate growth of the *Propionibacterium* and thereby enhance the growth-associated vitamin B_12_ biosynthesis [26, 33].

The addition of cobalt did not have an impact on vitamin B_12_ synthesis (Additional file 1: Table S2A). In addition, growth remained unchanged as well. Likewise, the extra addition of 50 µm CoCl_2_ to the MRS pre-culture medium to eventually pre-load the cells with cobalt for better performance did not reveal any stimulating effect (Additional file 1: Table S2B) neither did have the effect of higher concentrations of cobalt (Additional file 1: Table S2B). Obviously, the natural cobalt level of the plant milk (8 µg/kg) was sufficient. Supplementation of the milk with riboflavin (vitamin B_2_) increased vitamin B_12_ production by more than 20%, whereas DMBI and nicotinamide addition had no effect. Notably, lactate and glucose were found highly beneficial as extra carbon sources. Their addition resulted in 2.4-fold and 1.4-fold more vitamin B_12_, whereby growth of NCC 1177 was markedly increased. In contrast, a mixture of l-threonine, l-glutamate, glycine, and succinate, added at a low level to check for a growth-decoupled contribution to the B_12_ pathway itself, did not trigger vitamin B_12_ production. A combination of all supplements worked best. The mixture resulted in a more than five-fold increased vitamin B_12_ level, 12.3 µg (100 g^−1^) and much better growth, indicating additive effects (Additional file 1: Table S2A).

### Metabolic profiling unravels the lifestyle of *P. freudenreichii* NCC 1177 and its capabilities and inabilities in sunflower seed milk fermentation

**Fig. 3:**
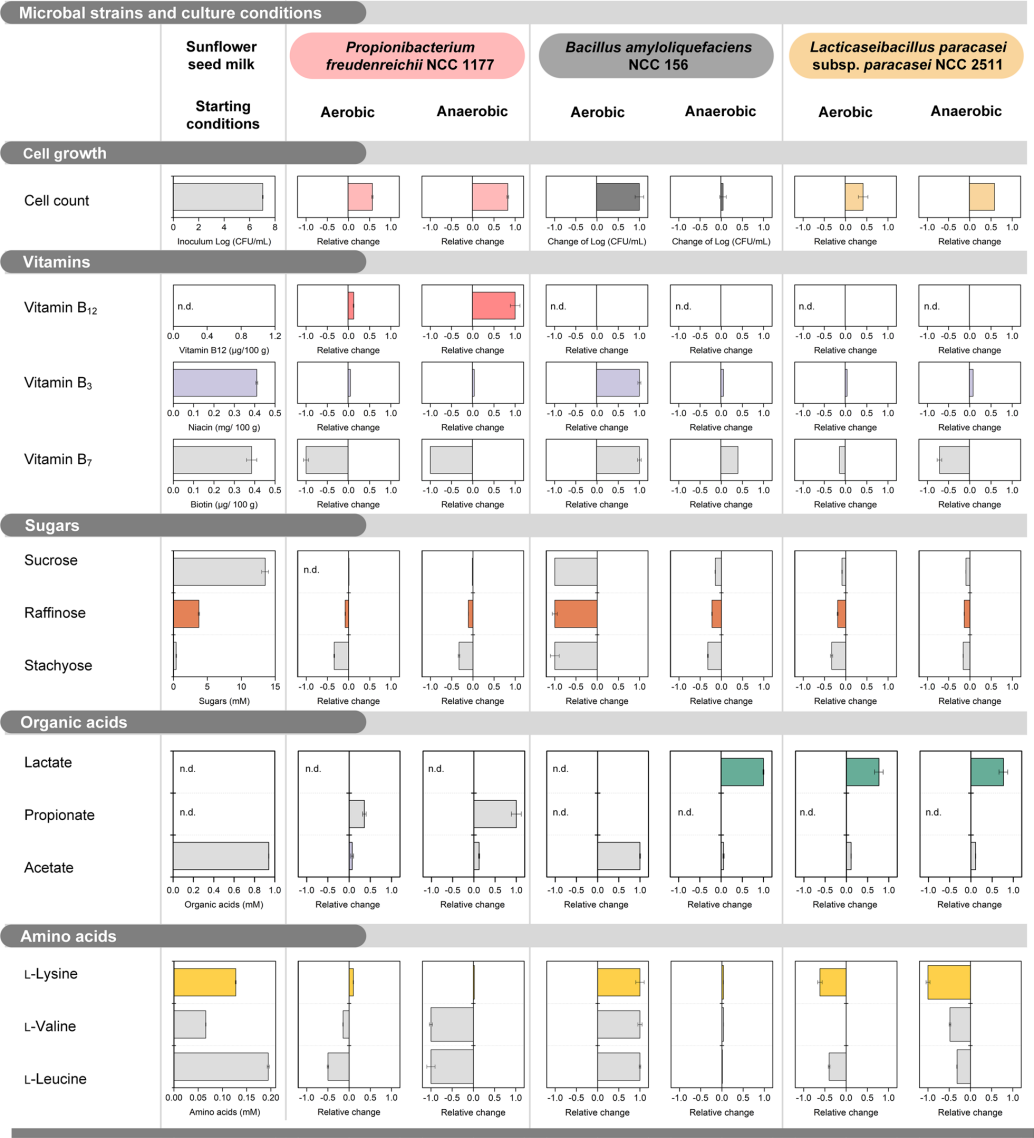
Metabolic signature profile of *P. freudenreichii* NCC 1177, *B. amyloliquefaciens* NCC 156, and *L. paracasei* subsp. *paracasei* NCC 2511 during aerobic and anaerobic cultivation of pasteurized sunflower seed milk. The data shown display the most relevant characteristics regarding growth, the content of vitamins, sugars, organic acids, amino acids, and flavor formation. The changes of metabolite levels are given as relative values. For each parameter, the maximum absolute concentration change (increase or decrease), observed among all conditions, was set to a value of 1. The changes of the other conditions were normalized to this maximum to allow a straightforward comparison. All corresponding absolute values are provided in Additional file 1: Table S3, whereby the maximum change, used for the normalization is highlighted. In addition, the starting values for non-fermented milk are shown. The fermentation was carried out at 30°C either anaerobically (48 h) or aerobically (24 h). n = 3

At this stage, it appeared important to study the lifestyle of strain NCC 1177 in more detail and identify the specific needs of the microbe. Cultures of the microbe in sunflower seed milk were conducted under (micro) aerobic (24 h) and under anaerobic (48 h) conditions and analyzed for cell growth and changes in various metabolites: sugars, organic acids, amino acids, and vitamins. Non-inoculated milk, incubated under the same conditions, served as a control. Under anaerobic conditions, NCC 1177 grew well from 10^7^ to almost 10^9^ cfu’s (Fig. 3, Additional file 1: Table S3). The strain formed 0.9 µg (100 g)^−1^ vitamin B_12_. Interestingly, the microbe had a strong demand for biotin (vitamin B_7_) but did not touch niacin (vitamin B_3_), also present. Furthermore, strain NCC 1177 did not efficiently consume the available sugars. It was not able to utilize sucrose, the most abundant sugar, and consumed only minor shares of raffinose and stachyose. Likewise, the acetate level remained largely constant. Lactate, an otherwise suitable carbon source was not available in the sunflower seed milk (Additional file 1: Table S3). Instead, growth of NCC 1177 seemed to rely on selected amino acids: l-isoleucine, l-alanine, l-serine, glycine, l-proline, and most strongly l-aspartate were consumed, well matching the catabolic amino acid spectrum of the microbe [34]. Propionic acid was observed as prominent by-product, and its occurrence nicely reflected the fermentative routes present in amino acid-grown propionibacteria [34, 35]. These microbes yield propionic acid from amino acids such as l-aspartate via the Wood-Werkman cycle to generate ATP and regenerate oxidized co-enzymes [36].

In comparison, growth under aerobic conditions was much weaker. Likewise, only a rather small amount of vitamin B_12_ was formed. Dissolved oxygen was available during the whole cultivation (Additional file 1: Fig. S2A). The amino acids l-aspartate and l-proline seemed the main growth substrates. Raffinose and stachyose were partially consumed, while sucrose and acetate were not used. Regarding other B vitamins, B_7_ was required and B_12_ was formed. The pH value remained relatively stable during the entire cultivation. One could conclude at this point that growth of the *Propionibacterium* in the presence of oxygen suffered from its weak capability to use the organic compounds in the sunflower seed milk as growth substrate. It goes without saying that anaerobic conditions were found optimal.

### *B. amyloliquefaciens *NCC 156 and *L. paracasei *subsp.* paracasei* NCC 2511 emerge as potential partners for co-cultures

We now wondered: could another microbe cover the specific needs of *P. freudenreichii* NCC 1177? Recently, *B. amyloliquefaciens* NCC 156 and *L. paracasei* subsp. *paracasei* NCC 2511 were identified as well-performing strains for chickpea milk fermentation [28]. Although this outcome was based on a different raw material, the multiple benefits discovered for the two strains (broad substrate range, production of essential amino acids, removal of indigestible sugars) appeared appealing to test them as potential partners for the *Propionibacterium*. To this end, they were both cultivated in sunflower seed milk, once under aerobic and once under anaerobic conditions.

*B. amyloliquefaciens* NCC 156 performed best under aerated conditions (Fig. 3, Additional file 1: Table S3). The strain exhibited strong growth (from 10^7^ to 10^9^ cfu’s) and efficiently utilized all major sugars—sucrose, raffinose, and stachyose. Interestingly, it accumulated significant amounts of vitamins B_3_ and B_7_ (Fig. 3). Furthermore, the microbe formed substantial levels of free amino acids (Additional file 1: Table S3). Due to the obviously high oxygen demand of *B. amyloliquefaciens* NCC 156, the dissolved oxygen level sharply decreased to 0% within 5 h and remained low until the end of the process (Additional file 1: Fig. S2C). The pH value dropped from 5.8 to 4.9 after 10 h and increased back to the starting value afterwards. Under anaerobic conditions, growth of NCC 156 was diminished. The strain fermented small amounts of sucrose (2.0 mM), stachyose (0.5 mM) and raffinose (0.1 mM), mainly into lactate (1.6 mM) and acetate (1.2 mM) (Additional file 1: Table S3). Vitamins were accumulated, although much less than in the aerated process.

In comparison *L. paracasei* subsp. *paracasei* NCC 2511 grew almost equally well under both conditions (Fig. 3, Additional file 1: Table S3). Under aerobic conditions, it degraded all three sugars and formed lactate as main by-product plus acetate. The pH value slightly decreased slightly from 5.8 to 5.5, whereas the dissolved oxygen level remained almost at full saturation (Additional file 1: Fig. S2B). Moreover, NCC 2511 exhibited a need for vitamin B_7_, and largely consumed free amino acids. The most pronounced differences, observed under anaerobic conditions, comprised a shift in substrate use from sugars to amino acids. Again, lactate and acetate were the major products formed.

Neither *B. amyloliquefaciens* NCC 156 nor *L. paracasei* subsp. *paracasei* NCC 2511, of course, formed vitamin B_12_ under any condition.

### Evaluation of double and triple co-cultures highlights *P. freudenreichii *NCC 1177 and *B. amyloliquefaciens* NCC 156 as perfect synergistic partners

As shown, the metabolic signatures of the different microbes appeared highly complementary. As example, *B. amyloliquefaciens* NCC 156 formed lactate, free amino acids, and vitamin B_7_, which were apparently all utilized by the *Propionibacterium* (Fig. 3, Additional file 1: Table S3). NCC 156 furthermore supplied vitamin B_3_, potentially stimulating B_12_ production (Additional file 1: Table S3). NCC 2511 formed lactate too. These patterns provoked an interesting question. Could co-culturing of *P. freudenreichii* NCC 1177 with the *Bacillus* and/or the *Lactocaseibacillus* enhance its growth and eventually boost vitamin B_12_ production? Therefore, *P. freudenreichii* NCC 1177 was co-cultured in LPP sunflower seed milk with each strain individually (double co-cultures) and with both strains together (triple co-culture). Like the initial studies, the 72-h cultures comprised two phases, an initial anaerobic phase (48 h), followed by an aerobic phase (24 h).

**Fig. 4:**
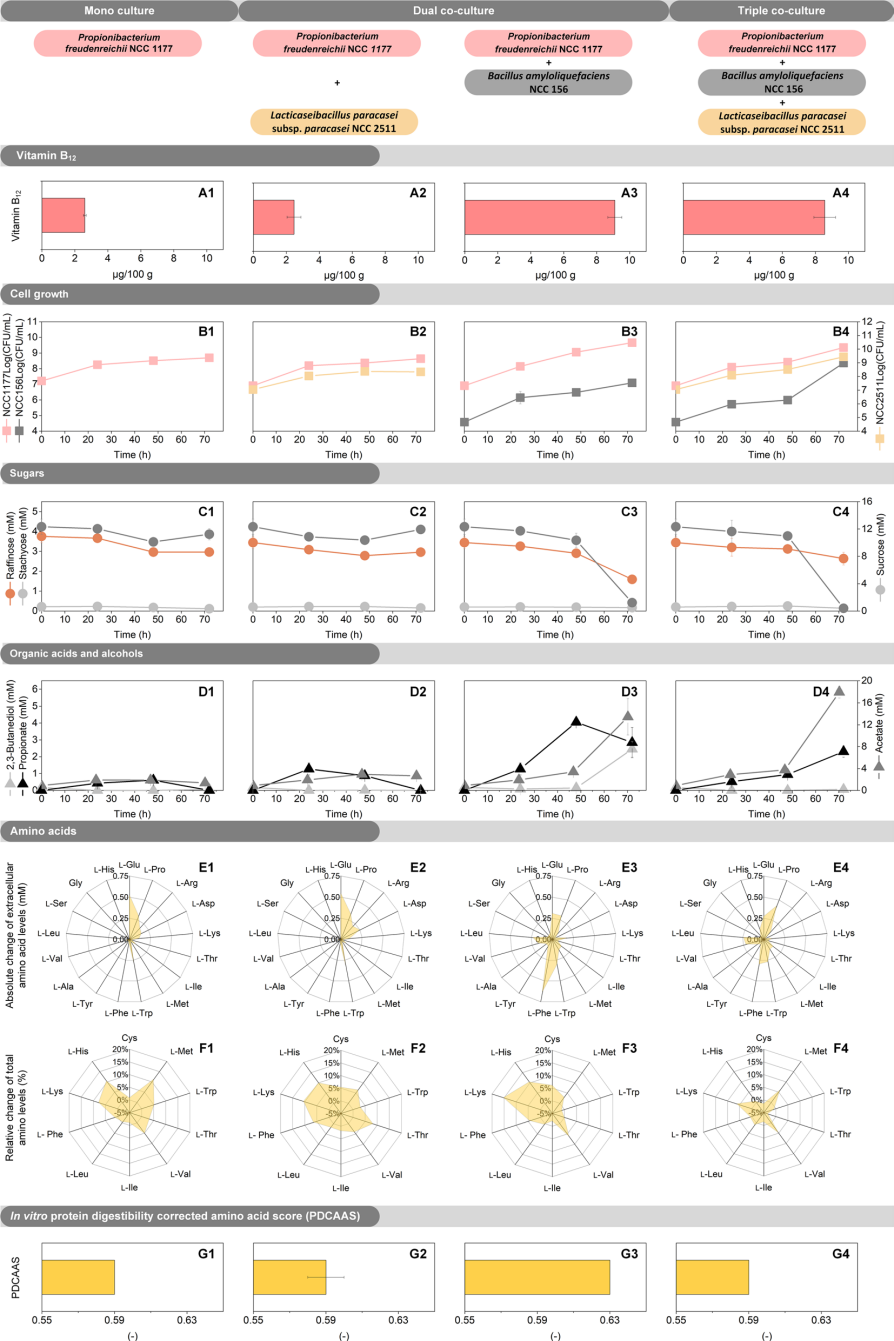
Performance of co-cultures on sunflower seed milk. The data comprise time-resolved changes for *P. freudenreichii* NCC 1177 (column 1), a dual co-culture of *P. freudenreichii* NCC 1177 and *L. paracasei* subsp. *paracasei* NCC 2511 (inoculated at 1:1 ratio, column 2), a dual co-culture of *P. freudenreichii* NCC 1177 and *B. amyloliquefaciens* NCC 156 (inoculated at 1000:1 ratio, column 3), and a triple co-culture of *P. freudenreichii* NCC 1177, *B. amyloliquefaciens* NCC 156, and *L. paracasei* subsp. *paracasei* NCC 2511 (inoculated at 500:500:1 ratio, column 4). Shown are vitamin B_12_ content (**A**), living cell number (**B**), sugar content (**C**), organic acid content (**D**), extracellular amino acid content (**E**), total amino acid content (**F**), and protein quality (**G**), expressed as in vitro protein digestibility corrected amino acid score (PDCAAS). The cultivation was carried out at 30 °C for 72 h, including an initial 48 h anaerobic phase, followed by a 24 h aerobic phase. n = 3

Grown together with *P. freudenreichii* NCC 1177, *B. amyloliquefaciens* NCC 156 improved vitamin B_12_ production by the *Propionibacterium* remarkably (Fig. 4A). In both co-cultures that contained the *Bacillus*, the final level of the vitamin was around 9 µg (100 g)^−1^, almost four-fold higher than in the fermentation with the *Propionibacterium* alone. The boost in vitamin B_12_ observed in the co-culture was almost as high as observed for NCC 1177 upon full supplementation with cobalt, various precursors, and biosynthetic stimulants (Additional file 1: Table S2A).

How did *B. amyloliquefaciens* NCC 156 boost vitamin B_12_ production? Clearly, it stimulated growth of the *Propionibacterium* during both phases of the fermentation. The latter achieved a tenfold higher cfu level by the presence of NCC 156 (Fig. 4B). This effect was observable in the double and the triple culture in which the strains were combined. First, NCC 156 formed lactate, a readily available carbon source for NCC 1177. Lactate itself was not detectable in the mixed cultures which let us to conclude that the entire amount that accumulated in anaerobic cultures of NCC 156 (Fig. 3, Additional file 1: Table S3), was completely re-consumed by the *Propionibacterium* in the co-culture. Second, NCC 156 provided a rich spectrum of free amino acids that displayed easily accessible carbon for the vitamin B_12_ producer (Fig. 4E). NCC 156 furthermore supplied vitamin B_3_, potentially stimulating B_12_ production (Fig. 3, Additional file 1: Table S3).

In addition, sunflower seed milk, fermented by the two-strain combination, was largely depleted of the indigestible sugars raffinose and stachyose (Fig. 4C, and the overall content of amino acids was substantially enhanced (Fig. 4F). The PDCAAS was 0.63, approximately 7% higher than that of all other fermented milks and that of the native plant milk (Fig. 4G).

Subsequent experiments aimed at optimizing the performance of this promising two-strain combination. Hereby, the inoculum ratio was identified as crucial parameter. A 1,000-fold excess of *Propionibacterium* over *Bacillus* cells resulted in a marked improvement of vitamin B_12_ production, whereby growth itself was not affected (Additional file 1: Table S4).

In contrast, *L. paracasei* subsp. *paracasei* NCC 2511 did not improve vitamin B_12_ production, when added. It neither stimulated the *Propionibacterium* directly nor provided an indirect benefit in the triple co-culture.

### Scaling the collaboration between *P. freudenreichii *NCC 1177 and *B. amyloliquefaciens* NCC 156 to pilot scale processed sunflower seed milk

Among all tested combinations, the co-culture of *P. freudenreichii* NCC 1177 and *B. amyloliquefaciens* NCC 156 seemed to work the best. It achieved the highest level of vitamin B_12_, the lowest level of indigestible sugars and the highest protein quality score—and thereby required only three things: natural sunflower seed milk and the two microbes. It was therefore selected for further optimization.

**Fig. 5:**
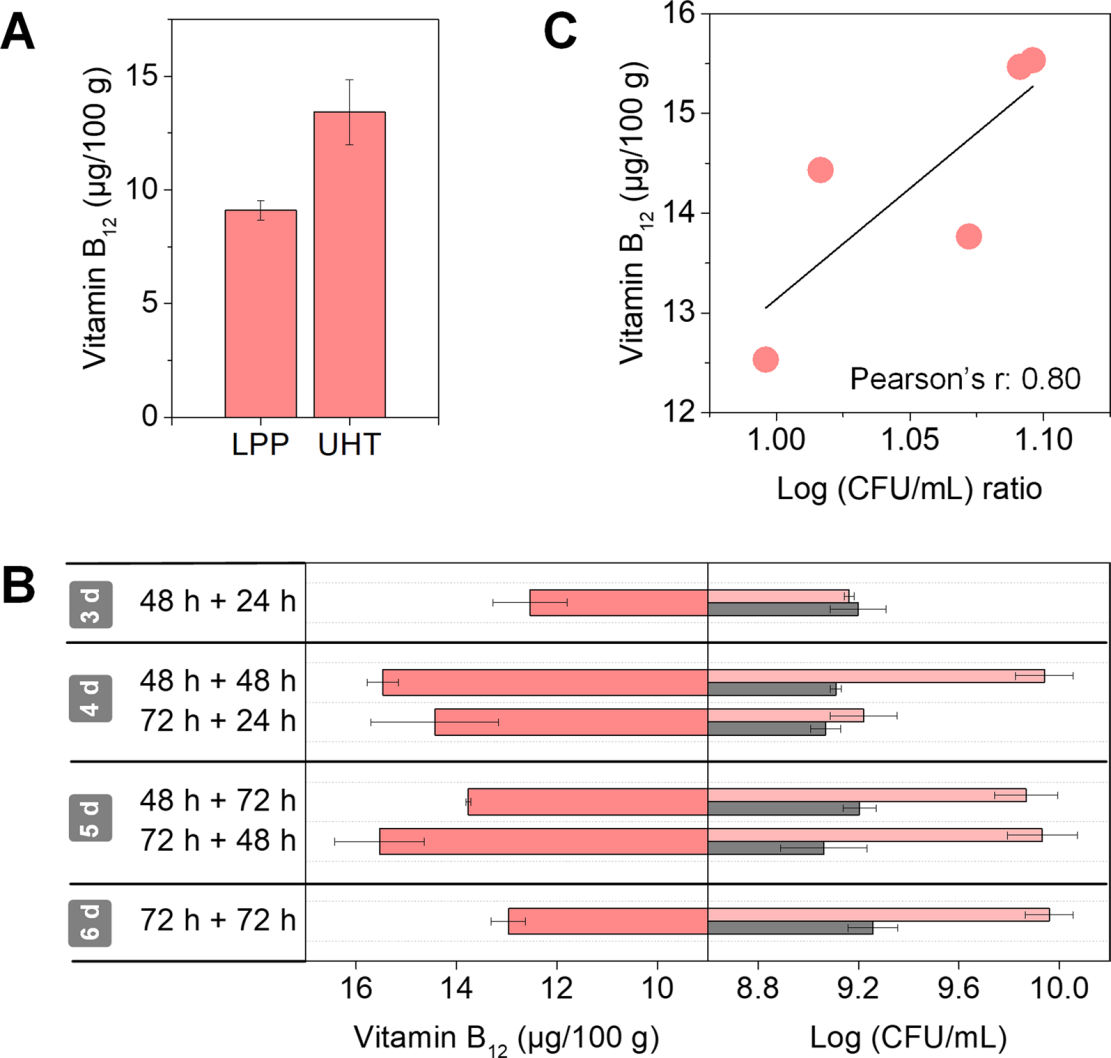
Impact of raw material pre-processing and inoculation ratio on the fermentation of sunflower seed milk by a co-culture of *P. freudenreichii* NCC 1177 and *B. amyloliquefaciens* NCC 156. Production of vitamin B_12_ on low pressure pasteurized (LPP) and ultra-high temperature (UHT) treated milk (**A**). Impact of the fermentation set-up with varied aerobic and anaerobic phases on cell growth and vitamin B_12_ production (**B**). Impact of the ratio between the strains on vitamin B_12_ production (**C**). For the latter, the numbers reflect the cfu ratio between NCC 1177 and NCC 156 at the end of the process. n = 3

The fermentation capacity of the co-culture of NCC 1177 and NCC 156 was now compared for the differently pre-treated milks, the LPP and the UHT processed one respectively, again using the workflow of three days (two days anaerobic and one day aerobic incubation). Notably, the co-culture performed excellent in UHT milk and yielded almost 40% more vitamin B_12_ than the lab scale LPP milk, i. e. a final level of 13 versus 9 µg (100 g)^−1^ (Fig. 5A). A possible reason might have been the more beneficial content of free amino acids by the UHT treatment (Additional file 1: Table S3 and S5).

### Making the collaboration between *P. freudenreichii *NCC 1177 and *B. amyloliquefaciens* NCC 156 more successful: tuning of aeration and fermentation time

**Fig. 6:**
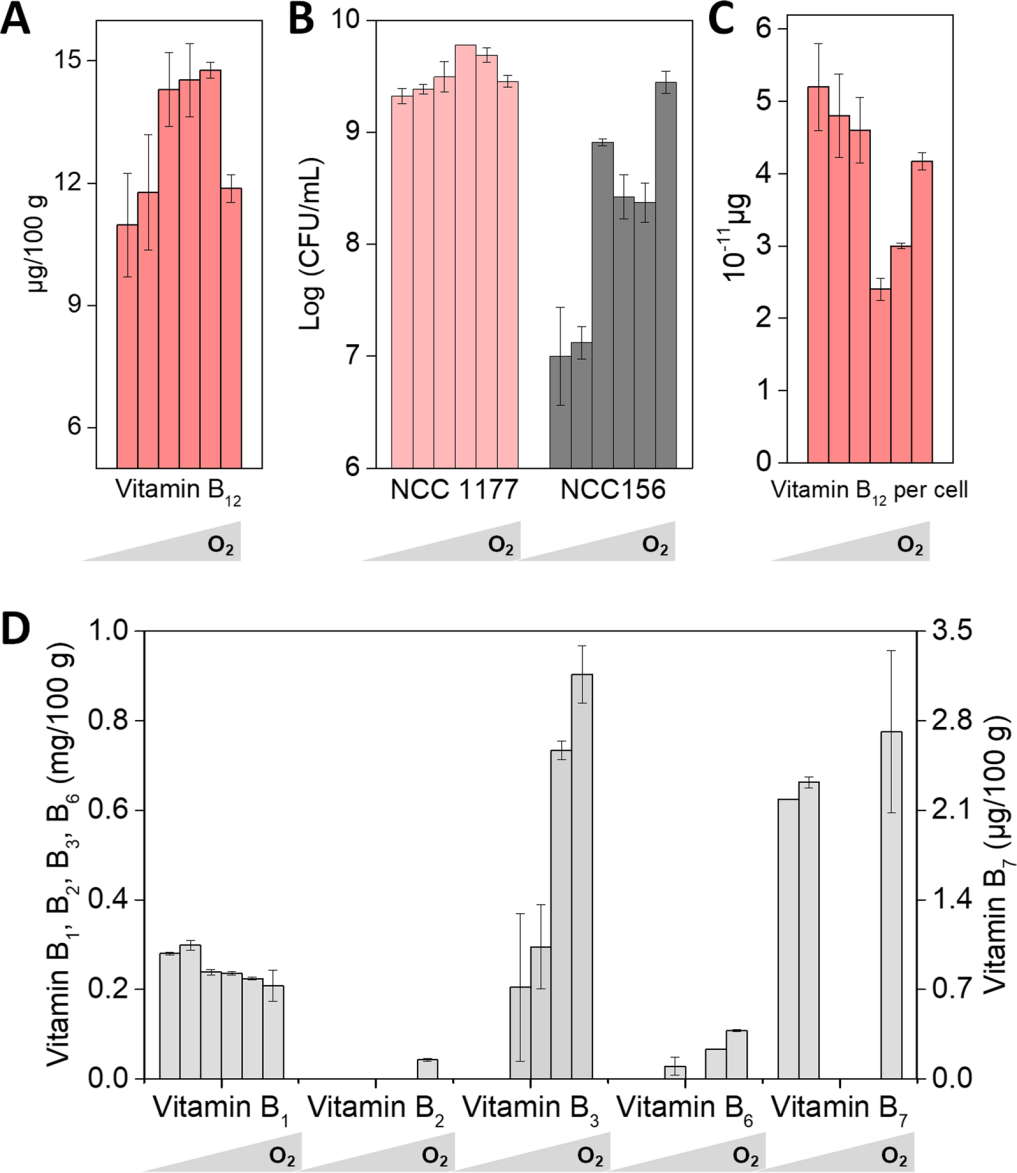
Impact of the dissolved oxygen level on the fermentation of sunflower seed milk by a co-culture of *P. freudenreichii* NCC 1177 and *B. amyloliquefaciens* NCC 156. Impact of oxygen supply on vitamin B_12_ production (**A**), growth (**B**), vitamin B_12_ production per cell (**C**), and the production of vitamins B_1_, B_2_, B_3_, B_6,_ and B_7_ during fermentation (**D**). Prior to fermentation, the milk was processed by ultra-high temperature treatment. The different aeration regimes from low to high (shown from left to right) were created by incubation in non-baffled flasks at 80 rpm, baffled flasks at 80 rpm, non-baffled flasks at 130 rpm, baffled flasks at 130 rpm, non-baffled flasks at 180 rpm and baffled flasks at 180 rpm. The inoculum ratio between strains NCC 1177 and NCC 156 was 1000:1. Fermentation was carried out at 30 °C for 72 h, including an initial 48 h anaerobic phase, followed by a 24 h aerobic phase. n = 3

Next, the influence of the dissolved oxygen level (during the second aerobic phase of the fermentation process) was evaluated. It turned out that a balanced oxygen supply was crucial to achieve a high vitamin B_12_ content (Fig. 6A). Too little aeration resulted in up to 26% lower titers, but too strong oxygen supply also reduced production. Furthermore, the oxygen supply influenced growth of both microbes, *B. amyloliquefaciens* NCC 156 and *P. freudenreichii* NCC 1177 (Fig. 6B), and the production of the vitamins B_2_, B_3_, and B_6_ (Fig. 6D), indicating a complex interplay. The specific vitamin B_12_ production per single cell, estimated from the data, revealed that NCC 1177 was most productive without oxygen exposure (Fig. 6C). However, also high oxygen supply resulted in good performance. Under these conditions, growth of *B. amyloliquefaciens* NCC 156 was strongest. The microbe supplied elevated levels of vitamins B_2_ and B_3_, known to stimulate B_12_ formation [14, 15]. Overall, the oxygen influence on vitamin B_12_ production appeared complex. It likely stimulated growth of NCC 1177 and NCC 156 but also activated the B_12_ biosynthetic pathway itself. One piece of the underlying complexity is the response of *P. freudenreichii* to oxygen. The microbe is known to be anerobic to aerotolerant [37], grows best anaerobically but is capable to generate energy also under microaerobic conditions using the TCA cycle and functional electron transport chains [38–41]. Due to the huge optimization potential of supplying the right amount of oxygen, it appears relevant to resolve this picture on the metabolic level in more detail in the future. Clearly, oxygen availability emerged as crucial factor to boost performance.

Optimum vitamin B_12_ production was as high as 14.8 µg (100 g)^−1^ and occurred under three different aeration regimes, indicating a robust process window. Finally, we tested different set-ups, regarding the duration of the process (Fig. 5B). A slightly prolonged aerobic fermentation phase allowed to increase vitamin B_12_ production even further. Notably, it the vitamin B_12_ level of the fermented sunflower seed milk appeared proportional to the inoculation ratio between *P. freudenreichii* NCC 1177 and *B. amyloliquefaciens* NCC 156 (Fig. 5C, r = 0.80). A dual phase fermentation with two days anaerobic and then two days aerobic incubation resulted in almost 16 µg (100 g)^−1^ vitamin B_12_. Similarly in performance was a three-plus-two-day setup.

### Metabolic interplay between *P. freudenreichii* and *B. amyloliquefaciens*

**Fig. 7:**
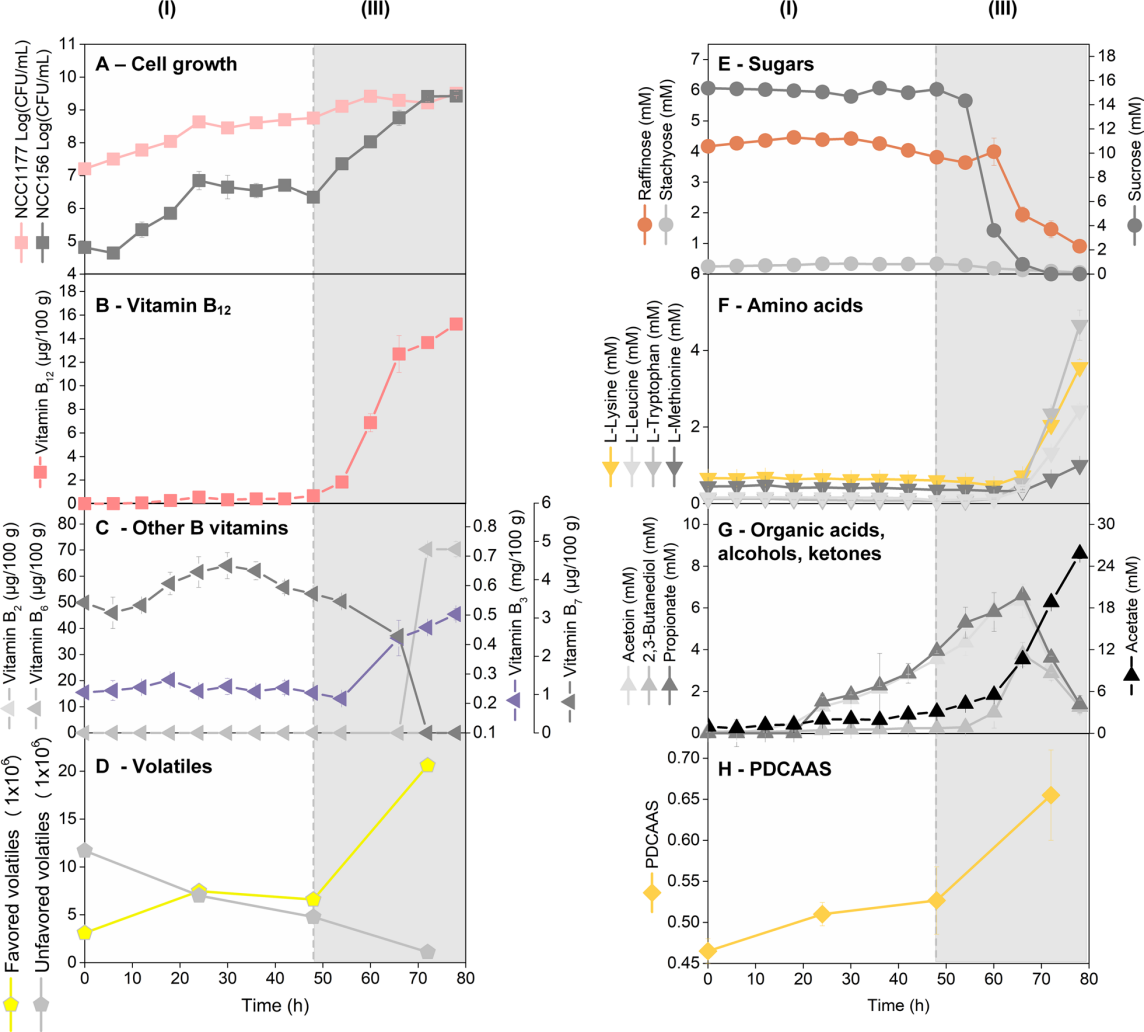
Dynamics of co-culture fermentation of *P. freudenreichii* NCC 1177 and *B. amyloliquefaciens* NCC 156 on UHT pre-treated sunflower seed milk. The data comprise living cell number (cfu) (**A**), vitamin B_12_ (**B**), vitamin B_3_, B_6_, and B_7_ (**C**), the area of favored and unfavored volatile (**D**), sucrose, raffinose, and stachyose (**E**), extracellular l-lysine, l-leucine, l-tryptophan, and l-methionine (**F**), acetoin, 2,3-butanediol, propionate, and acetate (**G**), PDCAAS (**H**). The phases of anaerobic (**I**) and aerobic (II) incubation are indicated in the time profile. The groups of favored and non-favored volatiles were assigned from the flavor properties of the individual compounds (see legend of Fig. 8). n = 3

The optimized process was now studied over 96 h, regarding the dynamics of strain growth, and the content of sugars, amino acids, organic acids, vitamins, and flavor compounds (Additional file 1: Fig. S3). The co-cultivation included a 2-day anaerobic phase, followed by a 2-day aerobic phase. Sunflower seed milk appeared best in composition after 80 h (Fig. 7). The initial anaerobic phase was the major growth phase of NCC 1177. After inoculation, the *Propionibacterium* immediately started to proliferate, and its cfu number increased about 30-fold over the first 48 h (Fig. 7A). Cells of NCC 156 multiplied almost 100-fold during the first 24 h. Notably, growth of NCC 156 resulted in an increased level of vitamin B_7_ (29.4%), which was afterwards consumed by NCC 1177 (Fig. 7C). In addition, the PDCAAS was slightly improved (14%) (Fig. 7H). Propionate (3.9 mM), acetoin (3.5 mM), and acetate (3.1 mM) were the major fermentation by-products during the anaerobic phase (Fig. 7G). An interesting picture resulted for the substrates. Sucrose was consumed from early on (by NCC 156, because NCC 1177 could not use it). Between 30 and 48 h, however, the sucrose level remained constant, while raffinose started to be consumed. The metabolization of this trisaccharide involves enzymatic cleavage into sucrose and galactose [42]. The constant sucrose level therefore resulted from a superposition of sucrose release from the cleavage of raffinose and (the probably) on-going sucrose consumption by NCC 156. Galactose was directly consumed, as it could not be detected. The sugar displays well-accessible carbon for *B. amyloliquefaciens* [43], explaining this observation. As shown for the monoculture, the fermentation of sucrose and galactose by *B. amyloliquefaciens* yielded lactate (Fig. 3). However, different to the monoculture, the organic acid was not observed in the co-culture. We conclude that lactate was immediately taken up as preferred carbon source by the *Propionibacterium*. In addition, the *Propionibacterium* used a range of amino acids during this stage (Additional file 1: Table S5).

Moreover, the anaerobic fermentation beneficially changed the flavor profile (Fig. 7D). With the onset of aeration after 48 h, the metabolism of the co-culture immediately changed. Growth of NCC 156 was strongly stimulated. NCC 1177 continued to grow, and the number of living cells increased once more about tenfold (Fig. 7A).

Notably, the aerobic phase was the major phase of vitamin B_12_ production. After 80 h, the vitamin had reached a high level of 15.2 (µg·100 g^−1^) (Fig. 7B). Notably, also other B vitamins were formed including vitamin B_3_ (0.5 mg·100 g^−1^) and vitamin B_6_ (70 µg·100 g^−1^) (Fig. 7C). Now, NCC 156 quickly consumed sucrose. From 60 h on, co-consumption of the disaccharide together with raffinose and stachyose was observed (Fig. 7E). When sucrose was depleted (66 h), propionate, acetoin, and 2,3-butanediol started to get consumed (Fig. 7G), while raffinose and stachyose degradation continued (Fig. 7E). The transient increase of the raffinose level seemed due to the onset of stachyose metabolism. Like raffinose, the tetrasaccharide stachyose belongs to the raffinose oligosaccharide family. It yields raffinose plus galactose during initial cleavage [42]. Regarding propionate, certain *Bacillus* strains can use it as a carbon source but *B. amyloliquefaciens* cannot [44]. In fact, the *Propionibacterium* re-used its own previously synthetized propionate, as also observed in the monoculture (Fig. 4). In the absence of other substrates, propionic acid is metabolized by *P. freudenreichii* using the reversed Wood–Werkman cycle [45]. This seemed the case here: NCC 1177 could not use the remaining sugars and lactate (one of its preferred carbon sources) was no more available, because NCC 156 did not form it under aerobic conditions (Fig. 4). Therefore, the *Propionibacterium* had to rely on organic acids or amino acids instead.

Overall, the co-fermentation showed an outstanding capacity to degrade indigestible sugars: raffinose (78.2%) and stachyose (78.8%) were largely depleted. Acetate (25.8 mM) was the major organic acid after 80 h. Remarkably, free extracellular amino acids, including l-lysine, l-methionine, and l-tryptophan, essential amino acids that are often limited in plant-based materials, increased during the final cultivation phase (Fig. 7F). This accumulation improved the PDCAAS to 0.66 (Fig. 7H). The amount of favoured flavor compounds (inferred from the total peak area during GC/MS analysis) increased three-fold during the aerobic phase, while unfavoured volatiles were reduced by 90.6% (Fig. 7D). A continuation of the fermentation process to finally 96 h, resulted in an even higher vitamin B_12_ level, 17.0 µg (100 g^−1^), as well as increased levels of vitamin B_2_, and extracellular amino acids. However, the prolongation of the process to 96 h somewhat decreased flavor value and the PDCAAS (Additional file 1: Fig. S3).

### Impact of fermentation on flavor development

**Fig. 8:**
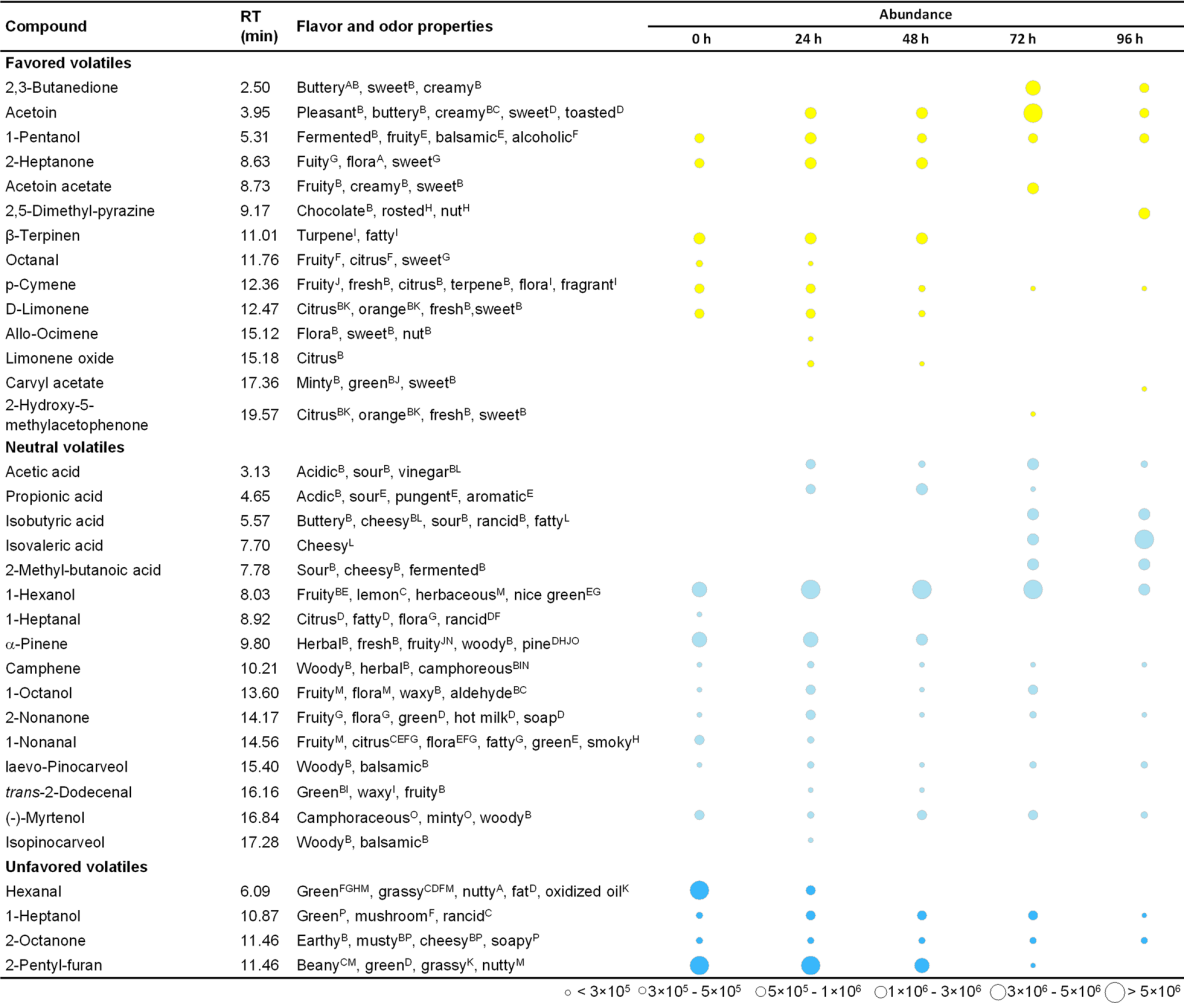
Flavor formation of food-grade microbes during sunflower seed milk fermentation. The data for flavor volatiles reflect the change in abundance in comparison to non-fermented sunflower seed milk (control). Classification into favored volatiles with flora, fruity, sweet, and creamy aroma properties (yellow), neutral volatiles with concentration dependent desired and non-desired aroma properties (light blue), and unfavored volatiles,potentially contributing to the beany, green, and mushroom flavor (dark blue) relates to previous dedicated studies and databases on odor and taste [82–97]. RT = retention time. The flavor properties are taken from previous studies and databases: A [82], B [83], C [84], D [85], E [86], F [87], G [88], H [89], I[90], J [91], K [92], L [93], M [94], N[95], O [96], P [97]. n = 3

GC–MS-based analysis revealed a strong impact of the co-culture on the spectrum of flavor related volatiles (Fig. 8). In total, 34 volatiles were identified in unfermented and fermented sunflower seed milk, including various saturated and unsaturated organic alcohols, aldehydes, ketones, organic acids, terpenoid, lactones, and furans. In unfermented sunflower seed milk, 1-hexanal and 2-pentyl-furan, which are volatiles with grassy and beany flavor, dominated, while sweet and fruity aroma compounds (e. g. 2-heptanone, β-terpinen, p-cymene, and D-lemonene) were present in lower amount. Co-cultures with *P. freudenreichii and B. amyloliquefaciens* changed the flavor profile significantly (Fig. 8). Several valuable volatiles were generated after 24 h, such as acetoin, 1-pentanol, allo-ocimene, limonene oxide, acetic acid, propionic acid, 1-octanol, 2-nonanone, trans-2-dodecanal, isopinocarveol, and 1-haptanol. After switching to aerobic conditions (48 h–72 h), acetoin and 2,3-butanediol, yielding buttery and fruity notes, were formed. Notably, the largest area of favoured volatiles was detected after 72 h fermentation. Unfavoured compounds such as hexanal and 2-pentyl-furan were completely removed after 48 h and 72 h of fermentation, respectively.

## Discussion

### Industrial impact of naturally fermented sunflower seed milk, rich in vitamin B_12_

As shown, co-fermentation of two carefully selected food-grade microbes provided sunflower seed milk which contained up to 17 µg (100 g)^−1^ vitamin B_12_, accompanied by concurrently increased levels of vitamins B_3_ and B_6_, improved protein quality and flavor profile, and strongly reduced amounts of indigestible sugars.

Vitamin B_12_ is one of the most important micronutrients for the human body but is unfavorably absent from plant-derived food [21], including sunflower seed milk. Therefore, vegetarians can face vitamin B_12_ deficiency regardless of their demographic characteristics, place of residency, age, and type of vegetarian diet [21]. Vitamin B_12_ deficiency can have severe consequences. It leads to increased homocysteine levels in the blood, a recognized risk factor for atherothrombotic and neuropsychiatric disorders [12, 21]. Of specific impact at this moment, vitamin B_12_, due to its various health benefits, seems to be a supporting active ingredient against COVID-19 symptoms and SARS-CoV-2 infections [46]. As example, methyl-cobalamin supplements help to reduce COVID-19-related organ damage and other symptoms [47]. A clinical study conducted in Singapore showed reduced COVID-19 symptoms in patients who received a daily supplementation with vitamin B_12_, vitamin D, and magnesium, reducing the need for oxygen and intensive care support [48].

Altogether, the demand for safe and cost-effective dietary nutrition with elevated vitamin B_12_ levels sharply increases. According to European Union food regulations, the recommended daily uptake (RDA) for vitamin B_12_ is 2.5 µg [49]. Hence, a single daily serving of 100 mL of our fermented sunflower seed milk would deliver up to sixfold of the RDA level for vitamin B_12_, far more than the minimum value required to claim: “high in content” (> 30% RDA). The vitamin B_12_ level [17 µg (100 g^−1^)] is among the highest values, achieved by supplement-free fermentation of plant-based materials. This achievement makes microbially co-cultured sunflower seed milk a promising alternative to animal-derived foods such as milk [0.4 µg (100 g^−1^)], eggs [1.4 µg (100 g^−1^)], lean red meat [3 µg (100 g^−1^)], and fish [2 µg (100 g^−1^)] [21] to provide vitamin B_12_.

### Fermentation provides sunflower seed milk with multiple benefits

Though numerous innovative food beverages from plant sources are being exploited as animal milk alternatives, many of these face some/any type of nutritional and organoleptic issues [2, 50]. Above all, the major reasons impeding the consumer’s interests and application of plant-based milks are: (i) inferior nutrient value limited by vitamin content and protein quality; (ii) poor digestibility due to the existence of indigestible compounds and anti-nutrients which may cause flatulence, diarrhoea, and other symptoms; (iii) off-flavor and taste such as beany, bitter, and earthy flavor associated to the raw materials. In this regard, appropriate multi-purpose strains and strain combinations appear crucial for upgrading these materials. It is therefore an important outcome of this study that *P. freudenreichii* NCC 1177 and *B. amyloliquefaciens* NCC 156 addressed several of the key requirements, partly alone but optimally in combination. Both strains showed robust growth in sunflower seed milk. *P. freudenreichii* NCC 1177 produced vitamin B_12_ (Additional file 1: Table S3), whereas *B. amyloliquefaciens* NCC 156 increased the l-lysine level and the overall protein quality, produced vitamins B_2_, B_3_, B_7_, and decreased indigestible sugars (Fig. 3). Likewise, strain NCC 156 provided several benefits in recent study on chickpea milk: increased levels of l-lysine, increased protein quality, decreased levels of indigestible sugars, and improved flavor profile [28], indicating that this strain works in different plant milks. Process control might help to prevent overgrowth of this fast-growing strain, where needed.

Here, the two well-performing isolates stand in a prominent line with related microbes, applied in plant-based fermentation and recognized as probiotics [23, 51]. Strains of *P. freudenreichii* were shown to synthetize vitamin B_12_ on plant-based materials such as barley (0.9–3.7 μg 100 g^−1^) [52], wheat (around 2.6–4.5 μg 100 g^−1^) [24, 53], durum (1.3 μg 100 g^−1^) [53], lupin (6.0 µg 100 g^−1^) [54], sauerkraut, and vegetable juice (7.2 μg 100 g^−1^) [55]. In addition, strains of *B. amyloliquefaciens* proved value regarding flavor formation and the hydrolysis of plant protein, the release of peptides, and the generation of bioactive compound and vitamins [56].

It is worth noting that as a monoculture, *Propionibacterium* needs a quite long fermentation time (up to 7 days or even longer) for optimized vitamin B_12_ production [22, 23, 26, 27]. In this study, we were able to shorten the fermentation time to 3 or 4 days, which is more efficient for industrial application. Moreover, our development comes with even more commercial benefits: improved flavor, improved digestibility, improved protein quality, and elevated levels of vitamins B_3_ and B_6_. The two strains NCC 1177 and NCC 156 are natural isolates, generally recognized as safe to be used in food fermentation[57]. Furthermore, no additives were used. The fermentation process contained only three ingredients: sunflower seed flour, the food-grade microbes, and water, enabling “clean labelling” as expected by consumers. Taken together, the developed co-culture fermentation displays a valuable development for human nutrition.

### *P. freudenreichii *NCC 1177 and *B. amyloliquefaciens* NCC 156 exhibit fine-tuned functional interactions

In the optimized co-culture fermentation process, several synergistic effects could be detected: (i) both strains showed better growth; (ii) vitamin B_12_ production of *P. freudenreichii* increased more than sevenfold as compared to mono-fermentation (17.0 µg 100 g^−1^); (iii) the PDCAAS was improved (29%), (iv) the level of l-lysine, the most limiting essential amino acid, was increased by 43%, (iv) the level of vitamins B_3_ (0.5 mg 100 g^−1^) and B_6_ (70 µg 100 g^−1^) was increased, and (v) the overall flavor profile was improved. Without doubt, the rich spectrum of benefits originated from microbial collaboration.

From a biochemical perspective, successful synthesis of vitamin B_12_ relies on several factors. First, and important to note here, vitamin B_12_ production in *P. freudenreichii* (including strain NCC 1177) is growth associated, making accessible and growth-promoting substrates one of the determinants [26]. Furthermore, cobalt and DMBI drive B_12_ production [27], while l-glutamate, glycine, l-threonine, and succinyl-CoA display the building blocks of the vitamin [58]. Earlier studies with *P. freudenreichii* cell homogenates showed that DMBI was derived from vitamin B_2_ (riboflavin) [59] and that the biosynthesis process was stimulated by vitamin B_3_ (nicotinamide) [15]. Here, we obtained a complex picture when trying out the effect of supplements on vitamin B_12_ production by NCC 1177 during sunflower seed fermentation. As shown, the addition of lactose, glucose, and riboflavin boosted vitamin B_12_ formation, while cobalt, DMBI, nicotinamide, l-glutamate, glycine, l-threonine, and succinate did not. These findings implied that major limiting factors were weak growth of *Propionibacterium* and low availability of vitamin B_2_. A combination of all supplements resulted in a more than five-fold increased vitamin B_12_ level, indicating the benefits of a more fine-tuned synergy regarding growth and supplementation.

On basis of the elaborated microbe’s physiology, growth and vitamin B_12_ biosynthesis were limited by several factors: (i) *P*. *freudenreichii* could only use extracellular amino acids but none of the sugars present in sunflower seed milk (Fig. 3); (ii) *P*. *freudenreichii* required micronutrients such as biotin to grow (Fig. 3) [60]; (iii) the microbe formed propionic acid causing an inhibiting pH decrease [61]; (iv) vitamin B_12_ biosynthesis was limited by insufficient availability of vitamin B_2_. Considering these requirements, *B. amyloliquefaciens* emerged as a perfect partner for *P. freudenreichii*, providing a natural dual culture consortium, co-working for improved growth, and finally yielding a multi-benefit fermentation. Proven interactions during the first phase included the donation of accessible carbon and essential micronutrients by NCC 156, stimulating growth of NCC 1177. During the second (aerobic) phase of the fermentation, *B. amyloliquefaciens* quickly proliferated. It released free amino acids from the protein and eventually lowered the dissolved oxygen level in a beneficial way, further stimulating growth of *P. freudenreichii.* In this regard, the ability of the *Bacillus* to form carbohydrases and proteinases and provide accessible carbon appeared crucial. At the same time, the *Bacillus* produced vitamin B_2_, vitamin B_3_, and vitamin B_7_, directly supporting vitamin B_12_ synthesis. We conclude that microbial cooperation more than compensates for a lack in key nutrients and adverse physicochemical conditions in plant-based materials, given that well-collaborating microbes are put together. Their careful selection and combination, as shown here, appears crucial for the success.

As shown, co-fermentation of NCC 156 and NCC 1177 was strikingly superior to that of the single strains. It stands in line with a range of successful co-fermentations, reported previously for food manufacturing, for example to produce yogurt, wine, and aroma-rich cocoa beans [62–64]. Co-fermentation of *P. freudenreichii* with the fungus *Rhizopus oryzae* on lupin tempeh yielded 20-fold more vitamin B_12_ than *P. freudenreichii* alone. Interestingly, the major interaction between these two microbes was hydrolysis of the seed protein by *R. oryzae*, releasing free amino acids to support growth of *P. freudenreichii* [54]. *Propionibacterium* also showed increased capacity to produce vitamin B_12_, when co-fermented with *Rhizopus oligosporus* on soybean [65], with kefir grains [66], and with different lactobacilli in wheat bran [24], soybean [65], and whey [25, 27, 67]. However, no synergistic effects on vitamin B_12_ production could be unraveled, when mixing *Propionibacterium* with lactobacilli [67].

## Conclusions

In this work, a genomic and metabolomic approach enabled the knowledge-based assembly of a consortium of food grade microbes for upgrading of sunflower seed milk, derived from sunflower press cakes as a waste product during sunflower oil production. This plant-based material offers the concept of sustainability and circular economy, when used for human consumption, and is therefore considered particularly eco-friendly in the strongly developing market of plant-based food [68]. As shown, the interactions between *B. amyloliquefaciens* NCC 156 and *P. freudenreichii* NCC 1177 enabled a co-operative process with remarkable benefits: (i) enriched content of vitamin B_12_, the key micronutrient for all vegans to be aware of, (ii) improved digestibility due to the removal of raffinose and stachyose, increased protein quality with increased levels of the most limiting essential amino acid l-lysine, and (iv) an improved flavor profile due the elimination of bitter notes and the generation of sweet and fruity aromas.

Notably, the key to the successful process was microbial collaboration. The excellent co-working of the strains even enabled a completely natural process without any supplementation. Inferred from the process data of the co-culture between 48 and 65 h (Fig. 7), a single cell of *Propionibacterium* synthetized up to 100 molecules of B_12_ per second which displays a remarkable synthetic power, considering the complexity of the vitamin.

Nature demonstrates the power of successful collaboration for complex tasks. Notably, the degradation of plant-based matter is mediated by microbial consortia with complementary features which even apply “task division strategies” [69]. This global principle, together with the outcome of this work, suggest looking more into microbial consortia for superior microbial food processing. Hereby, the understanding of the needs and capabilities of the microbes involved appears crucial to deliver food via transparent and natural processes, observed benefits as the results of natural processes catalyzed by safe microbes [70–73]. In this regard, it appears interesting to test the dynamic duo of NCC 156 and NCC 1177 on other plant-based milks.

## Material and methods

### Microorganisms

Strains of *Propionibacterium freudenreichii* (NCC 1124, NCC 1138, NCC 1145, NCC 1151, NCC 1159, NCC 1177, NCC 1186, NCC 1197, NCC 1216, NCC 1230, NCC 1236), *Lacticaseibacillus paracasei* subsp. *paracasei* NCC 2511, and *Bacillus amyloliquefaciens* NCC 156 were obtained from the Nestlé Culture Collection (NCC, Nestlé Research Centre, Lausanne, Switzerland). In addition, *P. freudenreichii* DSM 4902 and *P. freudenreichii* DSM 20,271 were obtained from the German Collection of Microorganisms and Cell Cultures (DSMZ, Braunschweig, Germany). All strains were classified as food-grade approved, based on the qualified presumption of safety (QPS) recommendation [57]. They were maintained as frozen stocks in 30% glycerol (v/v) at − 80 °C and are listed in Additional file 1: Table S1.

### Strain specific pre-culture and main culture media

Depending on individual nutrient requirements, different media were used for pre-cultivation of the different strains (Additional file 1: Table S1). All strains of *P. freudenreichii* and *L. paracasei* subsp. *paracasei* NCC 2511 and were grown in de Mann‐Rogosa‐Sharpe (MRS) medium, containing per litre: 52.0 g of MRS broth (Sigma-Aldrich, Steinheim, Germany) and 1.0 mL of Tween-80 (Sigma-Aldrich). In selected experiments, MRS medium was also used as main culture medium. In some of these studies, CoCl_2_ (50 µM) and dimethylbenzimidazole (DMBI, 100 µM) were added to the MRS medium [24, 74]. *B. amyloliquefaciens* NCC 156 was cultivated in modified tryptic soy broth (TSB) medium. It contained per litre: 17.0 g of tryptone (Becton Dickinson), 5.0 g of NaCl, 3.0 g of soytone (Becton Dickinson), 2.5 g K_2_HPO_4_, and 1.0 mL of 30% silicone antifoam (Sigma-Aldrich) [28].

### Differential agar media

Lactobacillus differential (LBD) agar was used to differentiate *P. freudenreichii* strains from *L. paracasei* subsp. *paracasei* NCC 2511. It contained per litre: 10.0 g of casein enzymatic hydrolysate (Becton Dickinson, Franklin Lakes, NJ, USA), 3.0 g of casein acid hydrolysate (Merck, Darmstadt, Germany), 1.5 g of enzymic digest of soybean meal (Becton Dickinson), 1.0 g of yeast extract (Becton Dickinson), 2.5 g of fructose (Amresco, Solon, OH, USA), 2.5 g of K_2_HPO_4_ (Sigma-Aldrich), 55 mg of bromocresol green (Sigma-Aldrich), and 15.0 g of agar (Becton Dickinson) [75]. In addition, TSB agar was used to grow *B. amyloliquefaciens* NCC 156 for the estimation of colony forming units. It contained per litre: 17.0 g of tryptone (Becton Dickinson), 5.0 g of NaCl, 3.0 g of soytone (Becton Dickinson), 2.5 g K_2_HPO_4_, and 1.0 mL of 30% silicone antifoam, and 15.0 g of agar (Becton Dickinson) [28].

### Low-pressure-pasteurized (LPP) sunflower seed milk medium

A suspension of sunflower seed press cake extract (6.8% w/w) was prepared by mixing 68 g of sunflower seed protein (All Organic Treasures) with 1 L of deionized water. The suspension was pasteurized at 90 °C for 6 h, while the temperature was monitored. Then, the milk was aseptically filled into sterile plastic bottles (2 L) and kept at 4 °C until use. Prior to cultivation, the LSP sunflower seed milk was manually homogenized. In selected supplementation studies, the pasteurized and homogenized sunflower seed milk was supplemented with one or several of the following additives: (i) CoCl_2_ (50 µM), riboflavin (40 µM), nicotinamide (27 mM), DMBI (100 µM), l-glutamate (0.6 mM), l-threonine (0.1 mM), glycine (0.3 mM), succinate (0.5 mM), lactate (1% w/w), and/or glucose (1% w/w). These were added from filter-sterilized stocks to final concentrations given above. Non-inoculated controls were incubated under the same conditions and evaluated for sterility by plating on TSB and LPD agar.

### Ultra-high-temperature (UHT) sunflower seed milk medium

A 6.8% (w/w) sunflower seed protein suspension was prepared by mixing 68 g sunflower seed protein (All Organic Treasures) with 1 L of deionized water. The suspension was homogenized and pre-heated to 75 °C, immediately followed by continuous ultra-high temperature (UHT) treatment in an automatized pilot-scale tubular heat exchanger (HT320 (ID920), OMVE, De Meern, The Netherlands). Hereby, the pre-warmed suspension was heated for 4 s to 143 °C at a flow rate of 30 L h^−1^ and then efficiently cooled down to 4 °C. All sterilization parameters were controlled by the integrated process control system. Finally, the milk was aseptically filled into sterile plastic bottles (2 L) and kept at 4 °C until use. Prior to fermentation, the UHT sunflower seed milk was manually homogenized. Non-inoculated controls were incubated under the same conditions and evaluated for sterility by plating on TSB and LPD agar.

### Pre-cultures

Strain specific settings (medium, temperature, and oxygen supply) were used to propagate pre-cultures (Additional file 1: Table S1) [28]. *P. freudenreichii* strains were grown anaerobically at 30 °C in 20 mL tubes, containing 10 mL MRS medium. For the first pre-cultivation, the tubes were inoculated from a glycerol stock (200 µL) and were then incubated overnight under a CO_2_ enriched atmosphere (9–13%) (Anaerobic atmosphere generation bags, Merck, Darmstadt, Germany). The first pre-culture of *L. paracasei* subsp. *paracasei* NCC 2511 was grown at 30 °C in 20 mL tubes, containing 10 mL MRS medium. For cultivation, the tubes were inoculated from a glycerol stock (200 µL), closed, and then incubated overnight, mimicking microaerobic conditions. *B. amyloliquefaciens* NCC 156 was grown aerobically. For this purpose, the microbe was cultivated overnight in 100 mL non-baffled shake flasks, filled with 10 mL modified TSB medium, inoculated from a glycerol stock (200 µL), and incubated on a rotary shaker (30° C, 130 rpm, 80% humidity, Infors, Bottmingen, Switzerland). Independent of the strain, cells from first pre-cultures were collected by centrifugation (5000 × *g*, 5 min, 4 °C) and used as inoculum for a second pre-culture, which was then grown overnight under the same conditions, then served as inoculum for the main culture. Seven selected strains were analysed for the correlation between optical density and colony forming units (cfu) during pre-cultivation. This yielded the following correlation factors *f* = cfu (OD_600_^−1^) during the exponential growth phase: 8.2E8 cfu (OD_600_^−1^) (NCC 1177), 5.3E8 cfu (OD_600_^−1^) (DSM 4902), 8.2E8 cfu (OD_600_^−1^) (NCC 1197), 1.9 E8 cfu (OD_600_^−1^) (NCC 1186), 3.8E8 cfu (OD_600_^−1^) (NCC 1138), 2.0E8 cfu (OD_600_^−1^) (NCC 2511), and 3.6 E7 cfu (OD_600_^−1^) (NCC 156). These factors were used to infer the required amount of pre-culture needed to prepare an inoculum of desired size (see below).

### Screening for vitamin B_12_ production

Strains of *P. freudenreichii* were grown in basic MRS medium and in supplemented MRS medium, additionally containing CoCl_2_ (50 µM) and DMBI (100 µM). Dual phase processes were conducted. Cultivation included a first anaerobic phase in 100 mL glass bottles (filled with 40 mL medium) which were inoculated from glycerol stocks and then incubated for 48 h in anaerobic jars under CO_2_ (9–13%). Afterwards, the broth was transferred to 100 mL baffled shake flasks and incubated aerobically for further 24 h on a rotary shaker (130 rpm, 80% humidity, 5 cm shaking diameter, Infors, Bottmingen, Switzerland). Three biological replicates were carried out for each condition.

### Sunflower seed milk processing

Prior to the process, the sunflower seed milk medium was manually homogenized. While cultivations were generally conducted at 30 °C in 6.8% sunflower seed milk, other conditions were varied for optimization and testing, as described above. Anaerobic incubations were done in 100 mL glass bottles, filled with 40 mL medium and incubated in anaerobic jars under CO_2_ (9–13%). Aerobic incubations were conducted in in 100 mL non-baffled shake flasks, filled with 40 mL medium and incubated on a rotary shaker (80% humidity, 5 cm shaking diameter, Infors, Bottmingen, Switzerland). The shaking rate was usually set to 130 rpm but changed in selected experiments as given above. Finally, dual phase setups were conducted. After a first anaerobic bottled phase in the jar system, the broth was transferred to a shake flask for a second aerobic phase. The incubation time was varied as given below. The processes were inoculated as follows. The optical density of the corresponding pre-culture was measured. The obtained correlation between optical density and cfu (see above) was then used to calculate the pre-culture volume that contained the desired colony forming units for inoculation. This volume was collected, followed by washing of the cells with water. Then, cells in the suspension were counted using a Neubauer counting chamber. On basis of the estimated cell concentration, an appropriate volume was then used as inoculum. The initial cell concentration was adjusted to 10^7^ cells mL^−1^, unless stated otherwise. In addition to monocultures, co-cultures were co-inoculated from individual pre-cultures in the same manner. Here, cells were added at different ratios, ranging from 1:1 to 1000:1, as given below. The corresponding conditions for each experiment are specified in the results section. Three biological replicates were carried out for each experiment. To minimize the impact of periodical sampling on culture conditions during time-resolved investigations, three complete incubations were sacrificed per data point, and evaporation of culture volume was considered for data correction, as previously described [72].

### Quantification of cell concentration

The cell concentration (OD_600_) was determined as optical density at 600 nm (UV-1600PC spectrophotometer, VWR, Hannover, Germany).

### Quantification of colony-forming units

Colony forming units (cfu) were determined by the plate serial dilution spotting method [28]. Briefly, 1 mL culture samples were sequentially diluted using 0.85% NaCl (w/v), supplemented with 1.0 g L^−1^ of tryptone (Becton Dickinson). For cfu estimation of *B. amyloliquefaciens* NCC 156, samples were spotted onto TSB agar. For cfu determination of *P. freudenreichii* and *L. paracasei* LPD agar was used. On this agar, the strain specific shape and colour of colonies formed allowed for a clear differentiation (Additional file 1: Fig. S1). All measurements were conducted in duplicate.

### Quantification of sugars, organic acids, and alcohols

Sugars were quantified by HPLC (Agilent 1260 Infinity Series, Agilent Technologies, Waldbronn, Germany), involving separation on a sulfonated spherical PS/DVB resin (VA 300/7.8 Nucleogel sugar Pb, Macherey–Nagel, Düren, Germany) and refractive index detection. Deionized water served as mobile phase at 80 °C and a flow rate of 0.4 mL min^−1^. Organic acids and alcohols were analysed by HPLC (Agilent 1260 Infinity Series, Agilent Technologies) using an ion-exchange column (Aminex HPX-87H, Bio-Rad, Hercules, CA, USA) as solid phase, 12 mM H_2_SO_4_ (0.5 mL min^−1^, 45 °C) as mobile phase, and refractive index detection (Hitachi, Tokyo, Japan). External standards were used for quantification.

### Quantification of vitamin B_12_

The analysis of vitamin B_12_ was based on the method proposed by AOAC International [76] which was slightly modified in this study [77]. Briefly, 3 mL sample was mixed with 2.5 mL sodium acetate solution (0.4 M, pH 4.0) and 100 µL cyanide solution (1% w/v in deionized water), and 4.4 mL deionized water. For vitamin B_12_ extraction, the mixture was incubated for 20 min at 107 °C. Solids were then removed by centrifugation (5000 × *g*, 30 min). A defined fraction of the obtained supernatant (3 mL) was subjected to an automated immunoaffinity clean-up (GX-271 ASPEC system, Gilson, Germany). The obtained eluate from the clean-up was evaporated to dryness and dissolved in 0.3 mL ultra-pure water (> 18.2 MΩ cm^−1^). Subsequently, vitamin B12 was quantified using HPLC (Waters Acquity UPLC, Waters, Milford, MA, US) with separation on a silica bonded column (Acquity UPLC® HSS T3, 1.8 µm, 100 × 2.1 mm, Waters) using a gradient of water and acetonitrile as mobile phase and UV detection (550 nm). External standards were used for quantification. Levels of vitamin B_12_ were given as (μg cyanocobalamin) 100 g^−1^.

### Thiamine, riboflavin, niacin, pyridoxine, and biotin analysis

Samples were hydrolysed in hydrochloric acid (100 °C, 35 min), neutralized, and filtered (0.22 µm, Millipore). The subsequent analysis was based on method LI-00.610 [78] using UHPLC-MS/MS with positive electrospray ionization and quantification via the peak area ratio of each analyte and an internal standard (Nestle Quality Assurance Center, Dublin, Republic of Ireland).

### Quantification of free amino acids

For the quantification of free amino acids, 1 mL sample was centrifuged (20,000 × *g*, 10 min, 4 °C). The obtained supernatant was filtered (0.22 µm, Millipore) and quantified by HPLC (Agilent 1100 Infinity, Agilent Technologies) including pre-column derivatization with *ortho*-phthaldialdehyde and α-aminobutyric acid as internal standard [79].

### Quantification of cobalt

The level of cobalt was quantified after sample work up by a high pressure asher (HPA-S High Pressure Asher, Anton Paar, IGZ Instruments, Zurich, Switzerland) using ICP-MS [78, 80].

### Estimation of sunflower seed milk protein digestibility and score

The nutritional quality of sunflower seed protein from non-fermented and fermented milk was assessed on the level of different parameters: in vitro protein digestibility (IVPD), amino acid score (AAS), and in vitro protein digestibility corrected amino acid score (PDCAAS) [81]. In short, IVPD, AAS, and PDCAAS were determined using the K-PDCAAS kit (Megazyme International, Bray, Co. Wicklow, Ireland), following the instructions of the manufacturer. Briefly, samples were sequentially digested by (i) pepsin under acidic conditions, (0.06 M HCl, pH 2), and (ii) trypsin/chymotrypsin under neutral conditions (1.0 M Tris, pH 7.4) to simulate the physiological conditions of gastric and intestinal digestion. Undigested protein was precipitated by the addition of trichloroacetic acid (40% w/v) and was then removed by centrifugation (15,000 × *g*, 10 min). The amount of reactive α-amino acids obtained after the treatment, was quantified after derivatization with ninhydrin into a purple dye via absorbance measurement at 570 nm. From the data, the IVPD was determined by correcting for the relative reactivity of certain α -amino acids (l-proline, l-lysine, l-histidine, l-arginine). The AAS was inferred from the ratio between the amino acid amount in the sample and the amount recommended by the FAO [31]. Finally, the PDCAAS was calculated by multiplying AAS and IVPD.

### GC–MS analysis of volatile flavor compounds

For the analysis of volatiles, headspace solid-phase micro-extraction (HS-SPME) (PAL RSI 120 autosampler, CTC Analytics, Switzerland) was coupled to GC–MS analysis (Agilent 8890 GC system, Agilent Technologies) [28]. Approximately 5 mL sample was immediately processed after collection. First, it was filled into a vial, supplemented with 1 g NaCl, and incubated for 20 min at 40 °C, and 400 rpm). Afterwards, an SPME fibre (65 µm, divinylbenzene/polydimethylsiloxane, preconditioned for 1 h at 260 °C, Agilent Technologies) was exposed to the headspace of the vial for 20 min to absorb the volatiles. Then, the fibre was placed into the GC–MS injector (300 °C, 3 min) for desorption [28]. The volatiles were then separated on an HP-5MS column (30 m, 0.250 mm, 0.25 µm, Agilent Technologies), using helium as carrier gas (0.4 mL min^−1^). Chromatograms were recorded by monitoring the total ion current (TIC) over a mass range from 30 to 300 m/z. Following deconvolution of the obtained signals (Agilent Chemstation, Agilent Technologies), individual analytes were identified through mass spectra library search (NIST/EPA/NIH Mass Spectral Library 08). The corresponding area counts for each compound were collected for quantification. Measurements were conducted in triplicate.

### Data processing and statistical analysis

All results displayed in Figures and Tables are shown as mean values ± standard deviation (SD). Statistical evaluation of the data was conducted by one-way analysis of variance (ANOVA). Differences in values were considered significant when the P value was less than 0.05 ( +) and 0.01 (+ +). Statistical analyses were performed by using SPSS (version 24.0).

## Supplementary Information

**Additional file 1: Figure S1.** Colony morphology used to assess strain-specific colony forming units in co-cultures. *P. freudenreichii* NCC 1177 on LPD agar (A); *B. amyloliquefaciens* NCC 156 on TSB agar; *L. paracasei subsp. paracasei NCC 2511 *on LPD agar (C). **Figure S2.** Time resolved changes of dissolved oxygen and pH value during aerobic growth on sunflower seed milk. The data comprise cultures using P*. freudenreichii* NCC 1177 (A), *L. paracasei subsp. paracasei NCC 2511 *(B),* B. amyloliquefaciens* NCC 156 (C), and a co-culture of two strains (D). n=1. **Figure S3.** Co-cultivation of *P. freudenreichii* NCC 1177 and *B. amyloliquefaciens* NCC 156 in UHT-processed sunflower seed milk. The data comprise colony forming units (A), the content of vitamin B_12_ (B), and vitamins B_3_, B_6_, and B_7_ (C), the relative amount of favored and unfavored volatile, inferred from the total peak area of GC/MS-based volatile analysis (D), the level sucrose, raffinose, and stachyose (E), the level of extracellular l-lysine, l-leucine, l-tryptophan, and l-methionine (F), the level of acetoin, 2,3-butanediol, propionate, and acetate (G), and the protein score PDCAAS (H). n=3. **Table S1.** Strain specific pre-culture conditions. As media, Mann-Rogosa-Sharpe medium (MRS) and modified tryptic soy broth (TSB) were used. Regarding oxygen supply, strains of P. *freudenreichii* were grown under anaerobic conditions. *L*. *paracasei* subsp. *paracasei* NCC 2511 was grown under microaerobic conditions, and *B. amyloliquefaciens* NCC 156 was grown aerobically. All strains were grown at 30 °C. **Table S2A.** Growth and vitamin B_12_ production of *P. freudenreichii* NCC 1177 on sunflower seed milk: Impact of different supplements added to the process. The incubation in the supplemented plant milk was carried out at 30 °C for 72 hours, including an initial anaerobic phase (48 hours), followed by an aerobic phase (24 hours). In addition, a non-supplemented process was conducted as control. The plant milk was pasteurized prior to cultivation. The vitamin B_12_ level and the cfu number reflect the final values at the end of the fermentation. n=3. **Table S3.** Metabolic profile of *P. freudenreichii* NCC 1177, *B. amyloliquefaciens* NCC 156, and *L. paracasei* subsp. *paracasei* NCC 2511 after aerobic and anaerobic growth on pasteurized sunflower seed milk. The fermentation was carried out at 30 °C either anaerobically (48 hours) or aerobically (24 hours). In addition, the composition of the milk at the start (including the inoculum) is given. For each parameter, the maximum absolute concentration change (increase or decrease), observed among all conditions, is highlighted in yellow. For the representation of the data as relative changes, this maximum change was normalized to a value of 1. The change of the other conditions was normalized to this maximum (Fig. 3). The data represent the final values under each condition. n=3. **Table S4.** Growth and vitamin B_12_ production during co-culturing of *P. freudenreichii* NCC 1177 and *B. amyloliquefaciens* NCC 156 in pasteurized sunflower seed milk: Impact of inoculum level and process conditions. In different set-ups, strain NCC 1177 was inoculated at a 10-fold, 100-fold, and 1,000-fold higher level than strain NCC 156. In all cases, the total inoculum of both strains was 2 × 10^7^ cfu mL^-1^. Regarding process operation, one set-up comprised first a 24 h aerobic phase, followed by a 48-h anaerobic phase, whereas the two phases were reverted in a second set-up. All fermentations were carried out at 30 °C. The plant milk was pasteurized prior to fermentation. Vitamin level and cell growth display the final values after 72 h. n=3. **Table S5.** Dynamics of free amino acids during co-culturing of *P. freudenreichii* NCC 1177 and *B. amyloliquefaciens* NCC 156 in UHT-treated sunflower seed milk. The process involved a 48-h anaerobic phase, followed by a 48-h aerobic phase. n=3.

## Data Availability

The dataset(s) supporting the conclusions of this article are all included within the article.
