# Supplementary material for: Co-cultures of Propionibacterium freudenreichii and Bacillus amyloliquefaciens cooperatively upgrade sunflower seed milk to high levels of vitamin B12 and multiple co-benefits
Source: Microb Cell Fact. 2022 Mar 26;21:48. doi: 10.1186/s12934-022-01773-w (PMC8959080; doi:10.1186/s12934-022-01773-w)
Supplement: Supplementary file 1 — Additional file 1: Figure S1. Colony morphology used to assess strain-specific colony forming units in co-cultures. P. freudenreichii NCC 1177 on LPD agar (A); B. amyloliquefaciens NCC 156 on TSB agar; L. paracasei subsp. paracasei NCC 2511 on LPD agar (C). Figure S2. Time resolved changes of dissolved oxygen and pH value during aerobic growth on sunflower seed milk. The data comprise cultures using P. freudenreichii NCC 1177 (A), L. paracasei subsp. paracasei NCC 2511 (B), B. amyloliquefaciens NCC 156 (C), and a co-culture of two strains (D). n=1. Figure S3. Co-cultivation of P. freudenreichii NCC 1177 and B. amyloliquefaciens NCC 156 in UHT-processed sunflower seed milk. The data comprise colony forming units (A), the content of vitamin B12 (B), and vitamins B3, B6, and B7 (C), the relative amount of favored and unfavored volatile, inferred from the total peak area of GC/MS-based volatile analysis (D), the level sucrose, raffinose, and stachyose (E), the level of extracellular l-lysine, l-leucine, l-tryptophan, and l-methionine (F), the level of acetoin, 2,3-butanediol, propionate, and acetate (G), and the protein score PDCAAS (H). n=3. Table S1. Strain specific pre-culture conditions. As media, Mann-Rogosa-Sharpe medium (MRS) and modified tryptic soy broth (TSB) were used. Regarding oxygen supply, strains of P. freudenreichii were grown under anaerobic conditions. L. paracasei subsp. paracasei NCC 2511 was grown under microaerobic conditions, and B. amyloliquefaciens NCC 156 was grown aerobically. All strains were grown at 30 °C. Table S2A. Growth and vitamin B12 production of P. freudenreichii NCC 1177 on sunflower seed milk: Impact of different supplements added to the process. The incubation in the supplemented plant milk was carried out at 30 °C for 72 hours, including an initial anaerobic phase (48 hours), followed by an aerobic phase (24 hours). In addition, a non-supplemented process was conducted as control. The plant milk was pasteurized prior to cultiva [file 12934_2022_1773_MOESM1_ESM.docx]

**Additional file to**

**Co-cultures of *Propionibacterium freudenreichii* and *Bacillus amyloquefaciens* cooperatively upgrade natural sunflower seed milk to high levels of vitamin B_12_ and multiple co-benefits**

*Microbial Cell Factories*

Muzi Tangyu^1^, Michel Fritz^1^, Lijuan Ye^2^, Rosa Aragão Börner^2^, Delphine Morin-Rivron^2^, Esther Campos-Giménez^2^, Christoph J. Bolten^2,3^, Biljana Bogicevic^2^, and Christoph Wittmann^1#^

^1^ Institute of Systems Biotechnology, Saarland University, Saarbrücken, Germany

^2^ Nestlé Research Center, Lausanne, Switzerland

^3^ Nestlé Product Technology Center Food, Singen, Germany

^#^Corresponding author: Phone/FAX: +49 681 302 71970/71972, e-mail: [christoph.wittmann@uni-saarland.de](mailto:christoph.wittmann@uni-saarland.de)

**
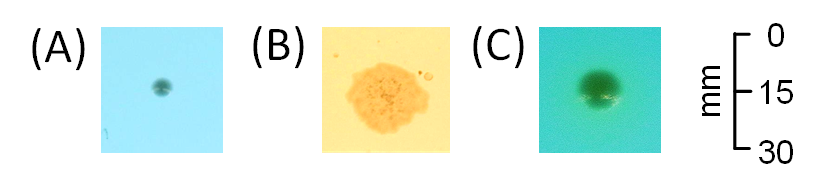
**

**Fig. S1: Colony morphology used to assess strain-specific colony forming units in co-cultures.** *P. freudenreichii* NCC 1177 on LPD agar (A); *B. amyloliquefaciens* NCC 156 on TSB agar; *L. paracasei subsp. paracasei NCC 2511* on LPD agar (C).


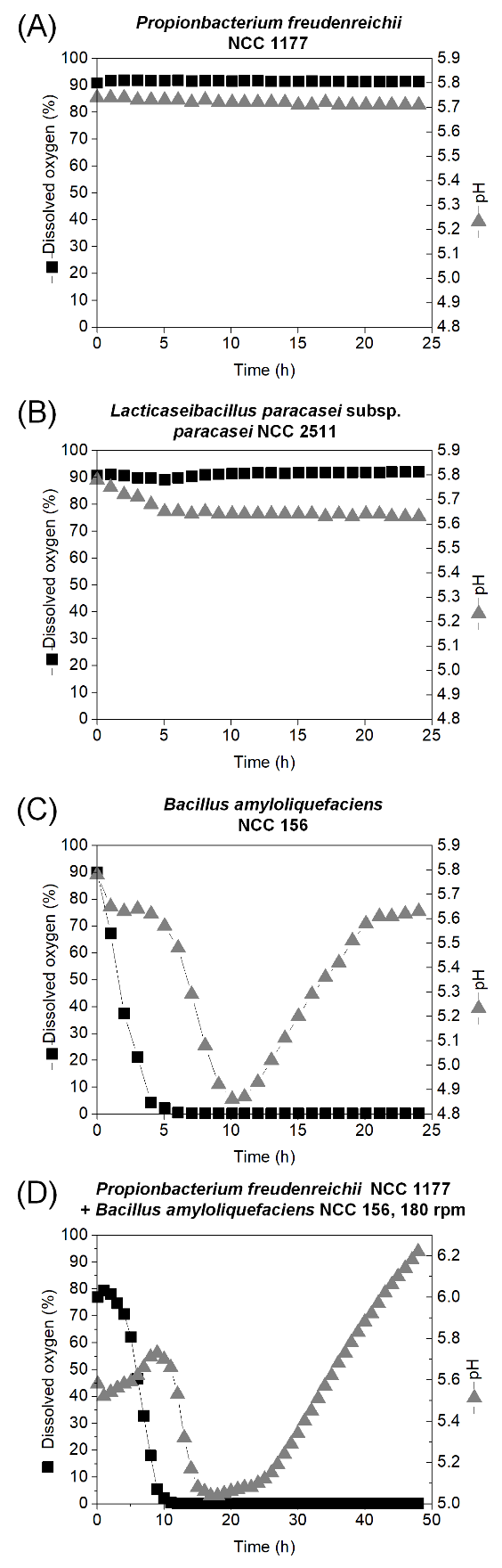


**Fig. S2: Time resolved changes of dissolved oxygen and pH value during aerobic growth on sunflower seed milk.** The data comprise cultures using P*. freudenreichii* NCC 1177 (A), *L. paracasei subsp. paracasei NCC 2511* (B), *B. amyloliquefaciens* NCC 156 (C), and a co-culture of two strains (D). n=1.


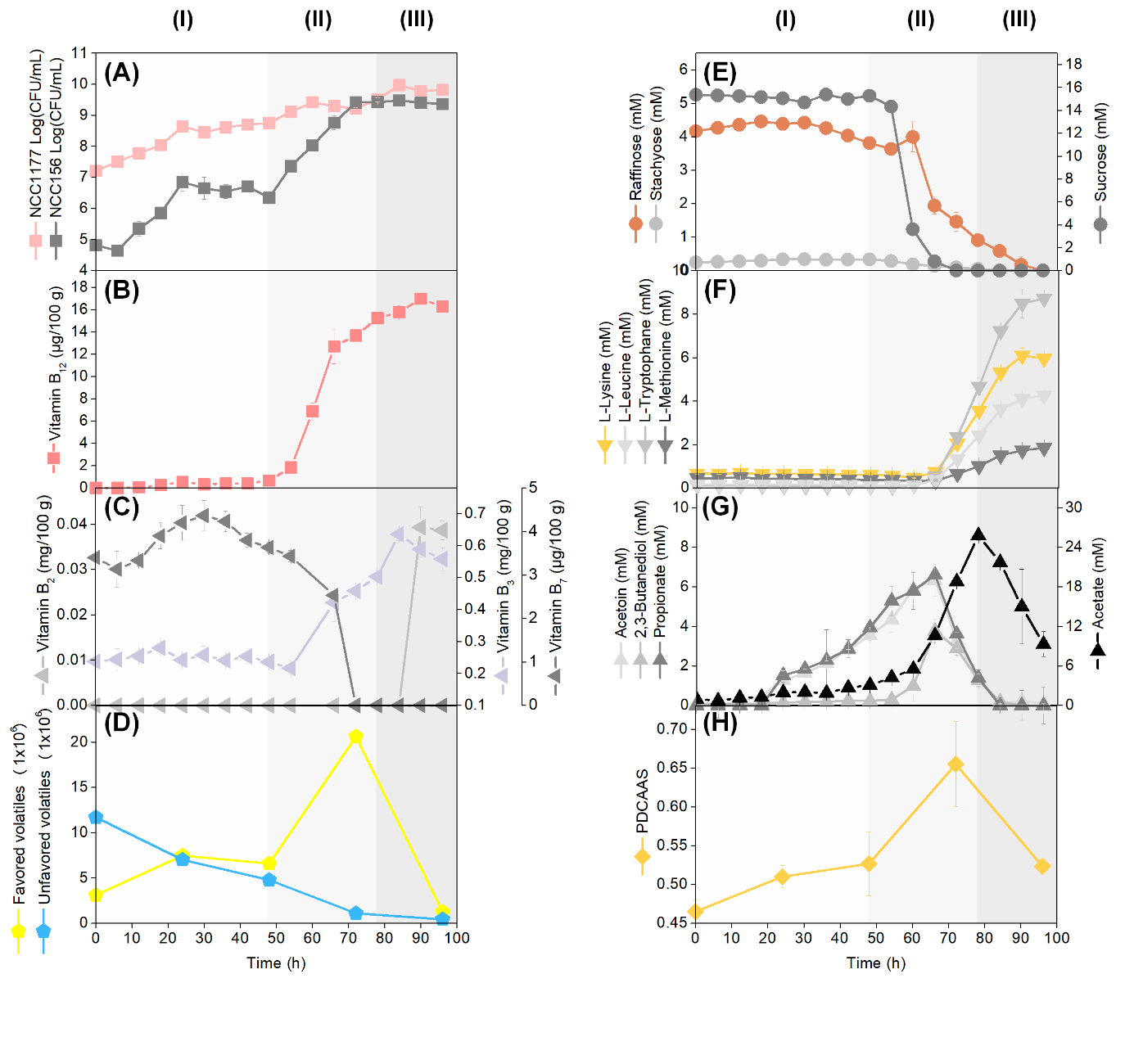


**Fig. S3: Co-cultivation of *P. freudenreichii* NCC 1177 and *B. amyloliquefaciens* NCC 156 in UHT-processed sunflower seed milk.** The data comprise colony forming units (A), the content of vitamin B_12_ (B), and vitamins B_3_, B_6_, and B_7_ (C), the relative amount of favored and unfavored volatile, inferred from the total peak area of GC/MS-based volatile analysis (D), the level sucrose, raffinose, and stachyose (E), the level of extracellular l-lysine, l-leucine, l-tryptophan, and l-methionine (F), the level of acetoin, 2,3-butanediol, propionate, and acetate (G), and the protein score PDCAAS (H). n=3.

**Table S1. Strain specific pre-culture conditions.** As media, Mann-Rogosa-Sharpe medium (MRS) and modified tryptic soy broth (TSB) were used. Regarding oxygen supply, strains of P. *freudenreichii* were grown under anaerobic conditions. *L. paracasei* subsp. *paracasei* NCC 2511 was grown under microaerobic conditions, and *B. amyloliquefaciens* NCC 156 was grown aerobically. All strains were grown at 30 °C.

| **Strains** | **Medium** | **Growth condition** |
| --- | --- | --- |
| *P. freudenreichii* NCC 1124 | MRS | Anaerobic |
| *P. freudenreichii* NCC 1138 | MRS | Anaerobic |
| *P. freudenreichii* NCC 1145 | MRS | Anaerobic |
| *P. freudenreichii* NCC 1151 | MRS | Anaerobic |
| *P. freudenreichii* NCC 1159 | MRS | Anaerobic |
| *P. freudenreichii* NCC 1177 | MRS | Anaerobic |
| *P. freudenreichii* NCC 1186 | MRS | Anaerobic |
| *P. freudenreichii* NCC 1197 | MRS | Anaerobic |
| *P. freudenreichii* NCC 1230 | MRS | Anaerobic |
| *P. freudenreichii* NCC 1236 | MRS | Anaerobic |
| *P. freudenreichii* DSM 4902 | MRS | Anaerobic |
| *L. paracasei* subsp. *paracasei* NCC 2511 | MRS | Microaerobic |
| *B. amyloliquefaciens* NCC 156 | TSB | Aerobic |

**Table S2A. Growth and vitamin B_12_ production of *P. freudenreichii* NCC 1177 on sunflower seed milk: Impact of different supplements added to the process.** The incubation in the supplemented plant milk was carried out at 30 °C for 72 hours, including an initial anaerobic phase (48 hours), followed by an aerobic phase (24 hours). In addition, a non-supplemented process was conducted as control. The plant milk was pasteurized prior to cultivation. The vitamin B_12_ level and the cfu number reflect the final values at the end of the fermentation. n=3.

|  | **Supplementation** | **Vitamin B_12_**  **(µg / 100 g)** | **Cell count**  **(log cfu / mL)** |
| --- | --- | --- | --- |
|  | No supplementation (control) | 2.4 ± 0.3 | 8.7 ± 0.1 |
|  | 50 µM CoCl_2_ | 2.1 ± 0.1 | 8.6 ± 0.0 |
|  | 40 µM vitamin B_2_ | 2.9 ± 0.4 | 8.8 ± 0.1 |
|  | 40 µM vitamin B_2,_ 27 µM vitamin B_3_ | 2.9 ± 0.1 | 8.7 ± 0.0 |
|  | 100 µM Dimethylbenzimidazole (DMBI) | 2.3 ± 0.3 | 9.0 ± 0.1 |
|  | 600 µM l-glutamate, 100 µM l-threonine, 300 µM glycine, 500 µM succinate | 2.2 ± 0.1 | 8.7 ± 0.0 |
|  | 1 % (w/w) lactate | 5.7 ± 0.2 | 9.4 ± 0.0 |
|  | 1 % (w/w) glucose | 3.4 ± 0.2 | 9.3 ± 0.0 |
|  | 50 µM CoCl_2,_ 40 µM vitamin B_2_, 27 µM vitamin B_3_, 100 µM DMBI 600 µM l-glutamate, 100 µM l-threonine, 300 µM glycine, 500 µM succinate, 1% (w/w) lactate | 12.3 ± 0.6 | 9.4 ± 0.0 |

**Table S2A. Growth and vitamin B_12_ production of *P. freudenreichii* NCC 1177 on sunflower seed milk: Impact of cobalt supplementation during preculturing of the microbe.** In short, the second pre-culture, conducted under anaerobic conditions in MRS medium, was supplemented with different levels of CoCl_2._ The main incubation in the supplemented plant milk was carried out at 30 °C for 72 hours, including an initial anaerobic phase (48 hours), followed by an aerobic phase (24 hours). In addition, a non-supplemented process was conducted as control. The plant milk was pasteurized prior to cultivation. The vitamin B_12_ level and the cfu number reflect the final values at the end of the fermentation. n=3.

|  | **CoCl_2_**  **(µM)** | **Vitamin B_12_**  **(µg /100 g)** | **Cell count**  **(log cfu / mL)** |
| --- | --- | --- | --- |
|  | 0 | 2.4 ± 0.3 | 8.7 ± 0.1 |
|  | 5 | 2.5 ± 0.1 | 8.7 ± 0.0 |
|  | 50 | 2.1 ± 0.1 | 8.8 ± 0.1 |
|  | 250 | 2.0 ± 0.1 | 8.9 ± 0.1 |

**Table S3. Metabolic profile of *P. freudenreichii* NCC 1177, *B. amyloliquefaciens* NCC 156, and *L. paracasei* subsp. *paracasei* NCC 2511 after aerobic and anaerobic growth on pasteurized sunflower seed milk.** The fermentation was carried out at 30 °C either anaerobically (48 hours) or aerobically (24 hours). In addition, the composition of the milk at the start (including the inoculum) is given. For each parameter, the maximum absolute concentration change (increase or decrease), observed among all conditions, is highlighted in Italics. For the representation of the data as relative changes, this maximum change was normalized to a value of 1. The change of the other conditions was normalized to this maximum (Fig. 3). The data represent the final values under each condition. n=3.

|  | **Sunflower seed milk** | | ***P. freudenreichii***  **NCC 1177** | | ***B. amyloliquefaciens***  **NCC 156** | | ***L. paracasei* subsp. *paracasei* NCC2511** | |
| --- | --- | --- | --- | --- | --- | --- | --- | --- |
|  | |  | Aerobic | Anaerobic | Aerobic | Anaerobic | Aerobic | Anaerobic |
| **Growth** | | | | | | | | |
| Cell count (log cfu mL^-1^) | | 7.07 ± 0.30 | 8.18 ± 0.03 | 8.69 ± 0.04 | *9.04 ± 0.20* | 7.15 ± 0.15 | 7.82 ± 0.22 | 8.15 ± 0.20 |
| **Vitamins** | |  |  |  |  |  |  |  |
| Vitamin B_12_ (µg 100 g^-1^) | | n.d.^a^ | 0.11 ± 0.01 | *0.90 ± 0.10* | n.d. | n.d. | n.d. | n.d. |
| Vitamin B_3_ (mg 100 g^-1^) | | 0.41 ± 0.01 | 0.42 ± 0.00 | 0.42 ± 0.01 | *0.64 ± 0.02* | 0.41 ± 0.01 | 0.41 ± 0.01 | 0.43 ± 0.01 |
| Vitamin B_7_ (µg 100 g^-1^) | | 3.85 ± 0.25 | 3.50 ± 0.22 | 3.50 ± 0.00 | *4.23 ± 0.19* | 4.00 ± 0.00 | 3.80 ± 0.00 | 3.60 ± 0.28 |
| **Sugars** | | | | | | | | |
| Sucrose (mM) | | 13.51 ± 1.13 | 13.65 ± 0.55 | 13.33 ± 0.51 | n.d. | 11.55 ± 0.44 | 12.36 ± 0.93 | 12.25 ± 0.44 |
| Raffinose (mM) | | 3.74 ± 0.32 | 3.54 ± 0.27 | 3.49 ± 0.11 | *1.38 ± 0.08* | 3.21± 0.10 | 3.29± 0.21 | 3.42 ± 0.12 |
| Stachyose (mM) | | 0.39 ± 0.03 | 0.29 ± 0.01 | 0.29 ± 0.02 | *0.07 ± 0.01* | 0.29 ± 0.01 | 0.29 ± 0.02 | 0.34 ± 0.01 |
| **Organic acids** | | | | | | | | |
| Lactate (mM) | | n.d. | n.d. | n.d. | n.d. | *1.64 ± 0.20* | 1.39 ± 0.11 | 1.26 ± 0.17 |
| Propionate (mM) | | n.d. | 0.48 ± 0.05 | *1.34 ± 0.16* | n.d. | n.d. | n.d. | n.d. |
| Acetate (mM) | | 0.94 ± 0.00 | 1.45 ± 0.66 | 2.43 ± 0.28 | *19.83 ± 0.24* | 1.17 ± 0.03 | 2.38 ± 0.20 | 2.22 ± 0.12 |
| **Amino acids** | | | | | | | | |
| l-Lysine (mM) | | 0.13 ± 0.00 | 0.15 ± 0.01 | 0.13 ± 0.00 | *0.39 ± 0.04* | 0.14 ± 0.01 | 0.12 ± 0.01 | 0.11 ± 0.01 |
| l-Valine (mM) | | 0.19 ± 0.00 | 0.17 ± 0.01 | 0.14 ± 0.01 | *0.74 ± 0.01* | 0.20 ± 0.02 | 0.17 ± 0.01 | 0.18 ± 0.00 |
| l-Leucine (mM) | | 0.07 ± 0.00 | 0.06 ± 0.00 | 0.04 ± 0.00 | *0.76 ± 0.04* | 0.09 ± 0.01 | - 1. ± 0.00 | 0.66 ± 0.00 |

^a^ n.d. – not detected

**Table S4. Growth and vitamin B_12_ production during co-culturing of *P. freudenreichii* NCC 1177 and *B. amyloliquefaciens* NCC 156 in pasteurized sunflower seed milk: Impact of inoculum level and process conditions.** In different set-ups, strain NCC 1177 was inoculated at a 10-fold, 100-fold, and 1,000-fold higher level than strain NCC 156. In all cases, the total inoculum of both strains was 2 × 10^7^ cfu mL^-1^. Regarding process operation, one set-up comprised first a 24 h aerobic phase, followed by a 48-h anaerobic phase, whereas the two phases were reverted in a second set-up. All fermentations were carried out at 30 °C. The plant milk was pasteurized prior to fermentation. Vitamin level and cell growth display the final values after 72 h. n=3.

| **Process**  **setup** | **Inoculation ratio**  **NCC 1177:NCC 156** | **Vitamin B_12_**  **(µg/100 g)** | ***P. freudenreichii***  **NCC 1177**  **(log cfu/mL)** | ***B. amyloliquefaciens***  **NCC 156**  **(log cfu/mL)** |
| --- | --- | --- | --- | --- |
| 24 h aerobic + 48 h anaerobic phase | 10:1 | 2.5 ± 0.2 | 8.9 ± 0.0 | 7.6 ± 0.1 |
|  | 100:1 | 3.5 ± 0.3 | 9.1 ± 0.2 | 7.0 ± 0.0 |
|  | 1000:1 | 3.8 ± 0.2 | 9.2 ± 0.1 | 6.7 ± 0.4 |
| 48 h anaerobic + 24 h aerobic phase | 10:1 | 6.3 ± 0.8 | 9.9 ± 0.1 | 9.3 ± 0.1 |
|  | 100:1 | 7.6 ± 0.5 | 10.0 ± 0.1 | 9.8 ± 0.1 |
|  | 1000:1 | 9.1 ± 0.4 | 9.7 ± 0.1 | 9.8 ± 0.1 |

**Table S5. Dynamics of free amino acids during co-culturing of *P. freudenreichii* NCC 1177 and *B. amyloliquefaciens* NCC 156 in UHT-treated sunflower seed milk.** The process involved a 48-h anaerobic phase, followed by a 48-h aerobic phase. n=3.

| **Anaerobic phase** | 0 h | 6 h | 12 h | 18 h | 24 h | 30 h | 36 h | 42 h | 48 h |
| --- | --- | --- | --- | --- | --- | --- | --- | --- | --- |
| Aspartate | 2.03±0.05 | 2.06±0.05 | 2.06±0.07 | 1.88±0.04 | 1.58±0.07 | 1.29±0.02 | 0.83±0.09 | 0.64±0.09 | 0.16±0.18 |
| Glutamate | 0.46±0.01 | 0.49±0.02 | 0.51±0.02 | 0.47±0.01 | 0.46±0.01 | 0.46±0.01 | 0.47±0.04 | 0.45±0.02 | 0.43±0.01 |
| Cystine | 0.40±0.01 | 0.41±0.00 | 0.41±0.01 | 0.38±0.03 | 0.37±0.01 | 0.37±0.00 | 0.36±0.00 | 0.36±0.01 | 0.37±0.01 |
| Serine | 0.18±0.01 | 0.17±0.00 | 0.18±0.01 | 0.17±0.01 | 0.15±0.02 | 0.14±0.01 | 0.14±0.01 | 0.12±0.01 | 0.10±0.00 |
| Histidine | 0.11±0.00 | 0.10±0.00 | 0.11±0.00 | 0.10±0.00 | 0.09±0.01 | 0.09±0.00 | 0.09±0.01 | 0.05±0.04 | 0.00±0.00 |
| Glycine | 0.34±0.00 | 0.34±0.01 | 0.33±0.01 | 0.29±0.01 | 0.23±0.02 | 0.19±0.02 | 0.12±0.01 | 0.13±0.01 | 0.11±0.00 |
| Threonine | 0.09±0.00 | 0.09±0.00 | 0.09±0.00 | 0.08±0.00 | 0.08±0.00 | 0.08±0.00 | 0.08±0.00 | 0.08±0.00 | 0.08±0.00 |
| Arginine | 0.42±0.01 | 0.42±0.02 | 0.44±0.02 | 0.40±0.01 | 0.39±0.04 | 0.38±0.01 | 0.38±0.03 | 0.36±0.01 | 0.33±0.01 |
| Alanine | 0.66±0.01 | 0.67±0.02 | 0.68±0.03 | 0.63±0.01 | 0.59±0.02 | 0.53±0.01 | 0.43±0.01 | 0.33±0.02 | 0.14±0.09 |
| Tyrosine | 0.06±0.00 | 0.06±0.00 | 0.07±0.00 | 0.06±0.00 | 0.06±0.00 | 0.06±0.00 | 0.05±0.00 | 0.05±0.00 | 0.04±0.00 |
| Valine | 0.27±0.01 | 0.28±0.01 | 0.28±0.01 | 0.26±0.00 | 0.25±0.02 | 0.24±0.00 | 0.22±0.02 | 0.19±0.00 | 0.15±0.01 |
| Methionine | 0.16±0.00 | 0.16±0.00 | 0.17±0.00 | 0.17±0.01 | 0.16±0.01 | 0.16±0.00 | 0.16±0.01 | 0.15±0.00 | 0.09±0.06 |
| Tryptophane | 0.45±0.02 | 0.45±0.03 | 0.49±0.03 | 0.41±0.02 | 0.42±0.01 | 0.40±0.02 | 0.42±0.05 | 0.39±0.02 | 0.36±0.01 |
| Phenylalanine | 0.15±0.00 | 0.15±0.00 | 0.16±0.01 | 0.14±0.01 | 0.13±0.01 | 0.12±0.00 | 0.12±0.01 | 0.11±0.00 | 0.10±0.00 |
| Isoleucine | 0.29±0.01 | 0.28±0.01 | 0.28±0.01 | 0.24±0.01 | 0.22±0.02 | 0.21±0.00 | 0.17±0.01 | 0.16±0.00 | 0.04±0.00 |
| Leucine | 0.12±0.00 | 0.13±0.00 | 0.13±0.00 | 0.11±0.01 | 0.09±0.01 | 0.09±0.00 | 0.08±0.00 | 0.08±0.00 | 0.07±0.00 |
| Lysine | 0.67±0.03 | 0.66±0.03 | 0.70±0.05 | 0.64±0.01 | 0.66±0.03 | 0.63±0.01 | 0.64±0.05 | 0.62±0.02 | 0.60±0.00 |
| Proline | 0.51±0.02 | 0.53±0.03 | 0.30±0.16 | 0.15±0.01 | 0.14±0.01 | 0.14±0.01 | 0.13±0.02 | 0.10±0.02 | 0.11±0.01 |

| **Aerobic phase** | 54 h | 60 h | 66 h | 72 h | 78 h | 84 h | 90 h | 96 h |
| --- | --- | --- | --- | --- | --- | --- | --- | --- |
| Aspartate | 0.03±0.00 | 0.03±0.00 | 0.19±0.20 | 1.28±0.14 | 0.93±0.07 | 1.21±0.05 | 1.18±0.07 | 1.09±0.07 |
| Glutamate | 0.04±0.00 | 0.10±0.03 | 0.52±0.09 | 2.11±0.23 | 3.54±0.30 | 4.46±0.30 | 4.60±0.19 | 4.49±0.29 |
| Cystine | 0.37±0.01 | 0.36±0.01 | 0.36±0.00 | 0.38±0.01 | 0.40±0.01 | 0.40±0.00 | 0.40±0.01 | 0.41±0.01 |
| Serine | 0.08±0.00 | 0.10±0.00 | 0.22±0.02 | 0.68±0.07 | 1.19±0.11 | 1.58±0.09 | 1.59±0.07 | 1.55±0.06 |
| Histidine | 0.00±0.00 | 0.11±0.00 | 0.30±0.05 | 1.05±0.08 | 1.91±0.18 | 3.04±0.11 | 3.52±0.18 | 3.36±0.06 |
| Glycine | 0.10±0.00 | 0.11±0.00 | 0.15±0.02 | 0.37±0.04 | 0.65±0.08 | 0.89±0.04 | 0.96±0.05 | 1.08±0.09 |
| Threonine | 0.05±0.00 | 0.06±0.01 | 0.17±0.02 | 0.56±0.08 | 0.83±0.09 | 1.08±0.07 | 1.11±0.06 | 1.10±0.05 |
| Arginine | 0.23±0.03 | 0.13±0.01 | 0.51±0.07 | 1.65±0.17 | 2.98±0.34 | 3.90±0.27 | 3.14±0.20 | 1.89±0.28 |
| Alanine | 0.12±0.01 | 0.16±0.01 | 0.31±0.02 | 0.78±0.08 | 1.25±0.09 | 1.56±0.11 | 1.59±0.04 | 1.53±0.07 |
| Tyrosine | 0.03±0.00 | 0.03±0.00 | 0.17±0.03 | 0.88±0.07 | 1.70±0.16 | 2.75±0.10 | 3.24±0.16 | 3.54±0.07 |
| Valine | 0.08±0.01 | 0.12±0.01 | 0.44±0.05 | 1.54±0.10 | 3.09±0.24 | 4.83±0.18 | 5.80±0.46 | 6.72±0.16 |
| Methionine | 0.11±0.00 | 0.13±0.00 | 0.37±0.04 | 1.32±0.10 | 2.43±0.17 | 3.63±0.13 | 4.09±0.22 | 4.25±0.06 |
| Tryptophane | 0.36±0.02 | 0.33±0.03 | 0.33±0.03 | 0.65±0.05 | 1.00±0.06 | 1.50±0.06 | 1.73±0.09 | 1.85±0.05 |
| Phenylalanine | 0.07±0.00 | 0.10±0.01 | 0.52±0.07 | 2.26±0.16 | 4.27±0.33 | 6.51±0.21 | 7.57±0.37 | 7.95±0.13 |
| Isoleucine | 0.10±0.00 | 0.11±0.01 | 0.29±0.03 | 1.01±0.07 | 1.96±0.17 | 2.91±0.09 | 3.32±0.22 | 3.59±0.09 |
| Leucine | 0.07±0.00 | 0.13±0.02 | 0.53±0.07 | 2.35±0.14 | 4.66±0.39 | 7.22±0.27 | 8.49±0.66 | 8.71±-0.08 |
| Lysine | 0.56±0.03 | 0.47±0.02 | 0.73±0.06 | 2.05±0.17 | 3.56±0.20 | 5.30±0.23 | 6.09±0.34 | 5.95±0.16 |
| Proline | 0.10±0.02 | 0.08±0.01 | 0.21±0.02 | 0.85±0.02 | 1.74±0.20 | 2.82±0.12 | 3.29±0.41 | 4.36±0.40 |
